# Recurrent somatic mutation and progerin expression in early vascular aging of chronic kidney disease

**DOI:** 10.1038/s43587-025-00882-6

**Published:** 2025-06-10

**Authors:** Gwladys Revêchon, Anna Witasp, Nikenza Viceconte, Hafdis T. Helgadottir, Piotr Machtel, Fabiana Stefani, Daniel Whisenant, Agustin Sola-Carvajal, Dagmara McGuinness, Nadia O. Abutaleb, Gonzalo Artiach, Emelie Wallén Arzt, Inga Soveri, Anne Babler, Susanne Ziegler, Rafael Kramann, Magnus Bäck, Anders Thorell, George A. Truskey, Lars Wennberg, Paul G. Shiels, Annika Wernerson, Peter Stenvinkel, Maria Eriksson

**Affiliations:** 1 Department of Medicine, Huddinge, Karolinska Institutet, Huddinge, Sweden; 2 Department of Clinical Science, Intervention and Technology, Division of Renal Medicine, Karolinska Institutet, Stockholm, Sweden; 3 Institute of Cancer Sciences, University of Glasgow, Glasgow, UK; 4 Duke University, Durham, NC USA; 5 Department of Medicine, Translational Cardiology, Karolinska Institutet, Stockholm, Sweden; 6 Department of Medical Sciences, Uppsala Universitet, Uppsala, Sweden; 7 Division of Nephrology and Clinical Immunology, University Hospital RWTH Aachen, Aachen, Germany; 8 Department of Clinical Sciences, Danderyd Hospital, Karolinska Institutet, Stockholm, Sweden; 9 Department of Surgery, Ersta Hospital, Karolinska Institutet, Stockholm, Sweden; 10 Department of Clinical Science, Intervention and Technology, Division of Transplantation Surgery, Karolinska Institutet, Huddinge, Sweden

**Keywords:** Genomic instability, Ageing, Experimental models of disease, Senescence, DNA damage and repair

## Abstract

Early vascular aging plays a central role in chronic kidney disease (CKD), but its molecular causes remain unclear. Somatic mutations accumulate in various cells with age, yet their functional contribution to aging tissues is not well understood. Here we found progerin, the protein responsible for the premature aging disease Hutchinson–Gilford progeria syndrome, steadily recurring in vascular smooth muscle cells of patients with CKD. Notably, the most common progeria-causing mutation, *LMNA* c.1824C>T, was identified as a somatic mutation in CKD arteries. Clusters of proliferative progerin-expressing cells in CKD arteries and in vivo lineage-tracing in mice revealed clonal expansion capacity of mutant cells. Mosaic progerin expression contributed to genomic damage, endoplasmic reticulum stress and senescence in CKD arteries and resulted in vascular aging phenotypes in vivo. These findings suggest that certain somatic mutations may be clonally expanded in the arterial wall, contributing to the disease-related functional decline of the tissue.

## Abstract (web summary)

Progerin, a truncated version of lamin A, causes Hutchinson–Gilford progeria syndrome characterized by premature aging and cardiovascular complications. Here the authors show that the most common progeria-causing mutation, *LMNA* c.1824C>T, is a somatic mutation in arteries from patients with chronic kidney disease, and that clonal propagation of the mutation in the vascular wall results in vascular aging phenotypes in mice.

## Main

Clonality of the arterial wall was first suggested in 1973 (ref. ^1^). Since then, several studies have confirmed the clonal nature of atherosclerotic plaques, and emerging evidence suggests that mutant vascular smooth muscle cells (VSMCs) expand clonally, promoting vascular disorders^1,2^. Recent technical advancements have made it possible to analyze somatic mutations in human progenitor cells and differentiated tissues. These revealed that somatic cells accumulate mutations during development and aging^3^. While many mutations are likely not functional, some may contribute to age-related disorders or become disease-causative when clonally expanded^4^. This somatic mutagenic process results in heterogeneous tissues composed of genetically variable cell clones, as observed in the esophagus, skin and liver^4,5^. Somatic mutagenesis has also been suggested to occur in the arterial wall where mutations in VSMCs were proposed to contribute to vascular diseases such as atherosclerosis^6,7^.

CKD is a widespread emerging public health priority (affecting 10–12% of the general population), leading to the progressive decline of kidney function. CKD is also associated with early vascular aging and an increased risk of cardiovascular disease (CVD), the main cause of mortality^8,9^. CKD arteries exhibit vascular abnormalities, including intimal thickening, VSMC loss and adventitia fibrosis (Supplementary Table 1). As a consequence of reduced renal function, patients with CKD present high levels of circulating uremic toxins, which are believed to lead to arterial stiffness and medial vascular calcification^10^. Traditionally, uremic vascular dysfunction is associated with an excessive production of reactive oxygen species in VSMCs or a reduced sensitivity of VSMCs to endothelium-derived vasorelaxing factors^11^. This suggests that the uremic environment present in patients with CKD plays a critical role in the development and progression of cardiovascular diseases; however, traditional risk factors do not fully explain such risk of developing CVD, highlighting the need for novel biomarkers predictive of CVD and improved targeted therapies.

Early vascular aging traits with similarities to that of patients with CKD can also be observed in patients with Hutchinson–Gilford progeria syndrome (HGPS) (Supplementary Table 1). HGPS is a rare genetic disease causing symptoms of accelerated aging in children. Affected patients are healthy at birth, and the disease symptoms manifest usually in the first years of life. HGPS children usually die in their early teens as a result of stroke or myocardial infarction^12^. Classic HGPS is caused by a germline mutation in a CpG dinucleotide in the *LMNA* gene, the c.1824C>T^13,14^. This mutation leads to the activation of a cryptic splice site and the production of a truncated lamin A protein termed progerin^13,14^. Lamins are major components of the nuclear lamina that provide structural support and have important roles in various cellular processes. In HGPS, progerin accumulates with age with a dominant negative effect and devastating impact on specific cells, including VSMCs calcification and promotion of atherosclerosis^15,16^. Progerin has also been found in various tissues, including arteries from non-HGPS individuals, with frequencies ranging from 0.001% to 0.09% in arterial media^17–22^, but the underlying mechanism, its functional relevance and contribution to age-associated diseases remain unknown.

Here, we hypothesized that progerin is involved in CKD-associated vascular aging. We identified progerin and the *LMNA* c.1824C>T mutation as a somatic event in CKD arteries and provide evidence for its clonal occurrence within the patients’ vascular wall. In vivo lineage-tracing showed that progerin-expressing VSMCs can clonally expand and contribute to molecular changes and phenotypes associated with early vascular aging in CKD. Our findings suggest that somatic mutations can potentially become a risk under chronic tissue damage, as seen in CKD.

## Results

### Clustered and scattered progerin-expressing cells in CKD arteries

**Fig. 1:**
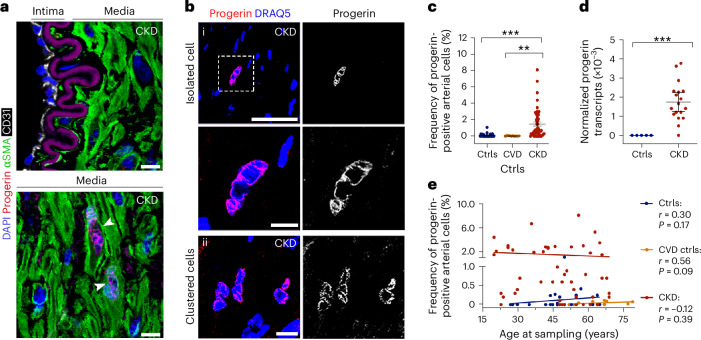
Progerin is recurrently expressed in up to 8.1% of the VSMCs in CKD arteries. **a**, Colocalization staining of progerin (red), CD31 (white) and αSMA (green) in CKD arteries. White arrowheads indicate progerin-positive cells. This staining was performed on five independent CKD arteries. **b**, Immunostaining on CKD arteries showing both isolated (**a**) and clustered (**b**) progerin-positive cells (red). **c**, Quantification of progerin-expressing cells in controls (*n* = 23), CVD controls (*n* = 10) and patients with CKD (*n* = 50) (CKD versus Ctrls: *P* = 3 × 10^−^^6^; CKD versus CVD Ctrls: *P* = 0.0012). **d**, Graph showing the copy number of progerin transcripts normalized to GAPDH, in controls (*n* = 5) and CKD (*n* = 18) arteries (*P* = 0.0003). **e**, Graph showing that the frequency of progerin-positive cells did not correlate with age at sampling. Scale bars, 20 μm (**a**), 50 μm (**b**(i)), 10 μm (**b**(ii)). Statistics were Kruskal–Wallis test with Dunn’s correction for multiple comparisons (**c**), Mann–Whitney test with a two-tailed 95% confidence interval (**d**), Spearman correlation coefficients with a two-tailed 95% confidence interval for the Ctrls (*n* = 23) and CVD Ctrls (*n* = 10) groups, and Pearson correlation coefficients with a two-tailed 95% confidence interval for the CKD group (*n* = 50) (**e**). Data are presented as mean values ± s.e.m. (**c**,**d**). **P < 0.01; ***P < 0.001. Source data

**Extended Data Fig. 1:**
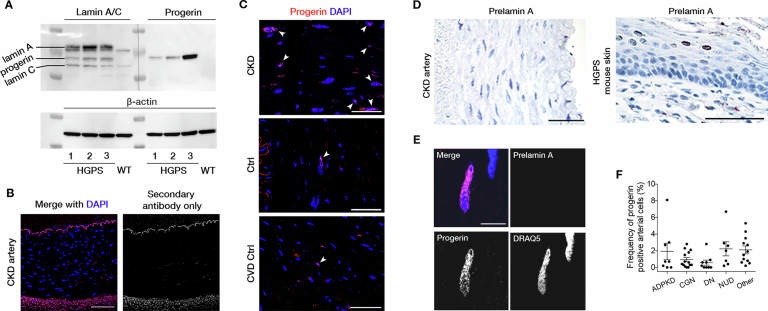
Detection of progerin, not prelamin A, in arteries from CKD patients. **(A)** Western blot on protein extracts from skin of HGPS and wild-type mice. The mouse model used is a transgenic model with a human *LMNA* minigene including the G608G mutation^50^. The lamin A/C antibody detected lamin A, progerin and lamin C (left), while the progerin antibody only detected progerin (right). **(B)** Representative picture of a negative control for an immunostaining against progerin in a CKD artery, which shows autofluorescence from the internal and external elastic lamina. **(C)** Immunofluorescence pictures of progerin (red) in CKD, Ctrl and CVD Ctrl arteries. White arrowheads point to progerin-positive cells. **(D)** Immunohistochemistry on CKD arteries using an antibody against prelamin A did not show positive cells, while prelamin A was detected in the skin of a HGPS mouse model^50^. This staining was performed on 14 CKD and 1 Ctrl artery. **(E)** Colocalization staining using progerin and prelamin A antibodies did not indicate the presence of prelamin A in progerin-positive cells. **(F)** Graph representing the frequency of progerin-positive arterial cells based on the etiology of CKD (n = 8 ADPKD, 14 CGN, 9 DN, 7 NUD, 12 Other); data presented as mean values ± SEM. Scale bars: B = 100μm; C-D = 50μm; E = 10μm. Source data

Epigastric arteries were obtained from 50 patients with CKD stage 5 (glomerular filtration rate (GFR) < 15 ml min^−1^) during living-donor renal transplantation. Control arteries were obtained from 34 individuals with or without history of CVD, hereafter referred to as CVD controls and controls, respectively. Basal characteristics of the samples are described in Methods and in Supplementary Table 2. Given the resemblance between the vascular pathology of patients with CKD and HGPS (Supplementary Table 1), CKD and control arteries were analyzed for progerin expression. We used an antibody known to detect progerin in both human cells and humanized mouse tissues^23–25^. This antibody recognizes progerin, but no other *LMNA*-derived proteins such as prelamin A, lamin A and lamin C^25^ (Extended Data Fig. 1a). Immunostaining of progerin with markers of endothelial cells (CD31) and VSMCs (αSMA) revealed the presence of progerin-expressing VSMCs in CKD arteries (Fig. 1a and Extended Data Fig. 1b). Progerin staining was exclusively nuclear and positive cells were not only isolated, scattered across the medial layer, but also forming clusters (Fig. 1b). Analysis of multiple sections per sample identified progerin-expressing cells in 82% of the patients with CKD. Progerin was found in up to 21.1% of the cells within the same section, its average frequency from multiple sections ranging between 0.1–8.1% (Fig. 1c, Extended Data Fig. 1c and Supplementary Table 3). Progerin-positive cells were also identified in the media of a few control and CVD control samples, although at significantly lower frequencies compared to CKD samples (Fig. 1c, Extended Data Fig. 1c and Supplementary Tables 4 and 5). Additionally, progerin expression was found at the RNA level in CKD arteries but not in control arteries (Fig. 1d and Supplementary Tables 3 and 4).

Investigation of coronary arteries previously showed higher frequencies of progerin-expressing cells in healthy aged individuals compared to healthy young individuals (age 1 month to 97 years)^19^. We observed no correlation with age in the present CKD population (age 20–69 years), nor in the control and CVD control populations (Fig. 1e). Earlier studies linked prelamin A to accelerated vascular aging^8^, but we did not detect its presence in CKD arteries (Extended Data Fig. 1d,e). As CKD has diverse causes, we compared progerin expression across different etiologies and found similar frequencies of progerin-positive cells, suggesting that its expression is independent of CKD origin (Extended Data Fig. 1f).

### *LMNA* 1824T is a somatic mutation in progerin-expressing arteries

**Fig. 2:**
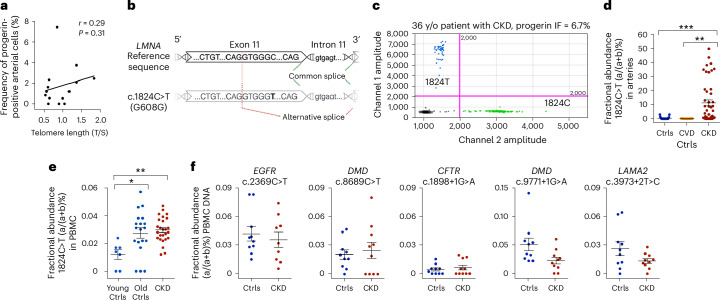
The common HGPS mutation, *LMNA* c.1824C>T, is a recurring somatic mutation in arteries of patients with CKD. **a**, Telomere length did not correlate with the frequency of progerin-positive cells in CKD arteries (*n* = 14). **b**, Schematic view of a region of the *LMNA* exon 11 containing the c.1824C>T mutation, which was shown to increase the usage of exon 11 cryptic splice site, resulting in the progerin splicing associated with HGPS. **c**, Example of a ddPCR two-dimensional plot showing the presence of the c.1824C>T mutation in a CKD artery with an allele frequency of 13.1% for the 1824T allele. This section had shown 6.7% progerin-positive arterial cells in immunofluorescence. IF, immunofluorescence. **d**, Graph showing the FA of the mutation in controls (*n* = 23), CVD controls (*n* = 9) and CKD arteries (*n* = 46) (CKD versus Ctrls, *P* = 3.6 × 10^−5^; CKD versus CVD Ctrls, *P* = 0.0035). **e**, Graph showing the FA of the mutation in young controls (*n* = 7), old controls (*n* = 19) and CKD (*n* = 26) PBMCs (young Ctrls versus old Ctrls, *P* = 0.0169; young Ctrls versus CKD, *P* = 0.0045). Young controls’ age range, 21–38 years; old controls’ age range, 44–81 years; patients with CKD age range, 20–38 years. **f**, Graphs showing the FA of five single nucleotide variants causing genetic diseases in Ctrls versus CKD PBMCs. These non-progeria genetic diseases include cystic fibrosis (*CFTR*), Duchenne muscular dystrophy (*DMD*), non-small cell lung cancer (*EGFR*) and congenital muscular dystrophy (*LAMA2*). Number of Ctrls and CKD individuals included, respectively: *EGFR* c.2369C>T: 10, 9; *DMD* c.8689C>T, *CFTR* c.1898+1G>A, *DMD* c.9771+1G>A, *LAMA2* c.3973+2T>C: 10, 10. Statistics were Spearman correlation coefficients with a two-tailed 95% confidence interval (**a**) and Kruskal–Wallis test with Dunn’s correction for multiple comparisons (**d**,**e**). Data are presented as mean values ± s.e.m. (**d**–**f**). *P < 0.05; **P < 0.01; ***P < 0.001. Source data

To understand the cause of progerin expression in CKD arteries, we first analyzed telomere shortening^22^ but found no correlation between reduced telomere length and progerin-positive cell frequency (Fig. 2a). As progerin expression in classic HGPS is caused by the *LMNA* c.1824C>T mutation (Fig. 2b), we next investigated whether it could drive progerin expression in CKD arteries. Using droplet digital PCR (ddPCR) on DNA from CKD and control artery sections, we detected the *LMNA* c.1824C>T mutation in 78.3% of the CKD arteries, with an average fractional abundance (FA) of 11.32%, suggesting that it is a somatic mutation. As the mutation was identified in progerin-positive sections, we can conclude that the somatic mutation is likely responsible for progerin expression in these arteries (Fig. 2c,d). In addition, the mutation FAs were significantly higher in the CKD arteries than in any of the control groups (mean 11.32% versus 0.43% versus 0.07% in CKD, controls and CVD controls, respectively) (Fig. 2d and Supplementary Tables 4 and 5). These data indicated that the *LMNA* c.1824C>T mutation is a hotspot mutation recurring in multiple individuals.

**Extended Data Fig. 2:**
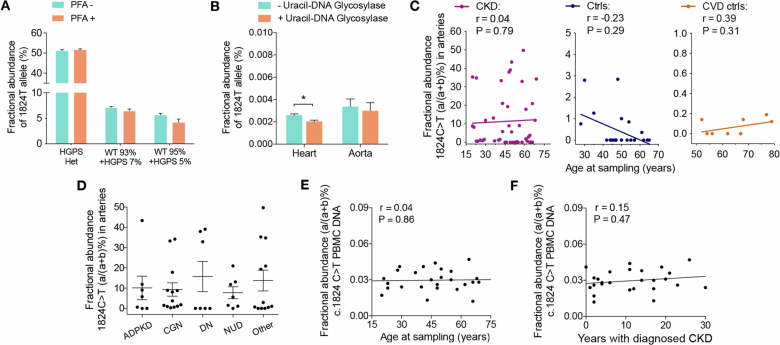
Very low PFA-induced mutations at the *LMNA* c.1824C locus. **(A)** Mosaic cell cultures composed of HGPS patient cells (carrying the *LMNA* c.1824C>T mutation) and control cells were treated with 4% PFA for 30 min. Analysis of the *LMNA* c.1824C and c.1824T alleles by ddPCR did not show allelic bias in PFA-treated vs. untreated cultures. **(B)** Uracil-DNA glycosylase treatment has previously been shown to reduce artefactual C > T transitions^60^. DNA extracted from >15-year-old, FFPE heart and aorta tissues of a mouse model with the wild-type human *LMNA* minigene^50^ were therefore treated with the enzyme (n = 3 Heart, n = 3 Aorta). ddPCR analysis revealed only very low, non-biological C > T transitions at position 1824 in heart (P = 0.0273), but not in aorta. **(C)** Graphs showing no significant correlation between age at sampling and the fractional abundance of the *LMNA* c.1824C>T mutation in CKD patients (left, n = 46), control individuals (middle, n = 23) and CVD controls (right, n = 9). **(D)** Graph representing the allele fraction of the *LMNA* c.1824C>T variant based on the etiology of CKD (n = 7 ADPKD, 13 CGN, 7 DN, 7 NUD, 12 Other). **(E-F)** Correlation graphs between the fractional abundance of the *LMNA* c.1824C > T in CKD PBMC and the age at sampling (**E**) or the number of years since CKD diagnosis (**F**)(n = 26). Statistics: **B**: Unpaired t-test with a two-tailed 95% confidence interval; **C** (CKD graph): Pearson correlation coefficients with a two-tailed 95% confidence interval; **C** (Ctrls and CVD Ctrls graph), **E**, **F**: Spearman correlation coefficients with a two-tailed 95% confidence interval. **A**, **B**, **D**: Data presented as mean values ± SEM. *, P < 0.05. Source data

Formaldehyde-based fixation was shown to result in low degree allelic imbalances^26^. Here, the detection of the c.1824C>T variant was performed on formalin-fixed arteries; however, the mutation was not detected in 26 out of 78 samples (Supplementary Tables 3–5). Formaldehyde-induced C:G to T:A mutations at the *LMNA* c.1824 locus were likewise negligible when tested in controlled experimental settings. Our results showed that only allele frequencies of the mutations at this locus ≤0.0047% could be accounted by formaldehyde activity or caused by other, nonbiological factors (Extended Data Fig. 2a,b).

Other studies have shown that there is an age-related accumulation of somatic mutations in different tissues, here there was no correlation between individual age and the c.1824C>T variant FA in the arteries (Extended Data Fig. 2c). The frequency of the mutation also seemed to be independent from the etiology of CKD (Extended Data Fig. 2d).

### The 1824T mutation is present at low allele frequency in blood

To test whether the *LMNA* c.1824C>T mutation was present in other tissues, we had access to peripheral blood mononuclear cells (PBMCs) from patients with CKD (26 out of 50) and 26 control individuals. Using ddPCR, we detected the somatic variant at low allele frequencies in all CKD PBMC samples (0.012–0.047%), and most PBMC control samples (0–0.057%) (Fig. 2e and Supplementary Table 3). Its FA increased with age in control PBMCs and showed a similar pattern in older controls and patients with CKD (Fig. 2e); however, there was no correlation between the *LMNA* variant FA and age or number of years between CKD diagnosis and collection of the arterial biopsy (Extended Data Fig. 2e,f). We also analyzed the occurrence of other mutations in different locations across the genome by ddPCR. These non-*LMNA* mutations are known to cause genetic diseases when present in the germline, with a similar effect on their respective protein as the *LMNA* mutation on the lamin A protein (Supplementary Table 6). FA analysis of these mutations showed no clear difference between CKD and control PBMC DNA samples (Fig. 2f). The presence of the mutation in blood, even though at low allele frequency, further emphasizes that the *LMNA* c.1824C loci is a mutational hotspot.

### Progerin correlates with duration and severity of vascular disease

**Extended Data Fig. 3:**
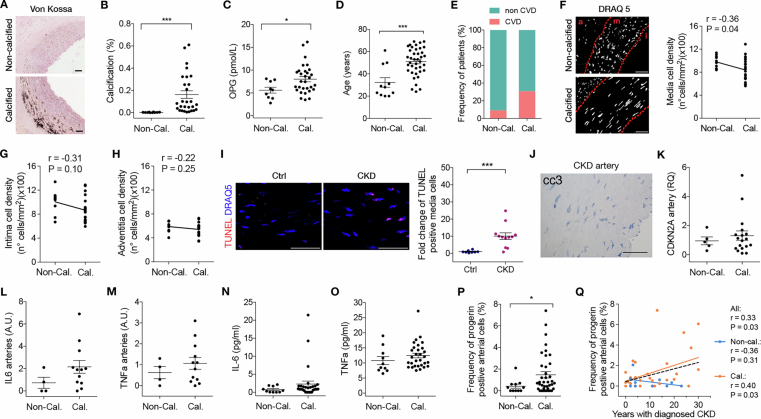
Progerin expression correlates with media calcification and the number of years with diagnosed CKD. (**A**) Von Kossa staining on CKD arteries. **(B)** Pathological vascular calcification assessment showed similar grouping as with semi-automated calcification scoring (n = 38 CKD, P = 1e^-8^). **(C)** Graph showing the circulating levels of osteoprotegerin (OPG) in CKD patients with non-calcified and calcified arteries (n = 39 CKD, P = 0.0356). (**D**) Arterial calcification was seen with increased age (n = 50 CKD, P = 0.0005). (**E**) Graph representing the frequency of patients with CVD based on arterial calcification. (**F**) Pictures showing the cell density in non-calcified and calcified arteries from CKD patients (left; a=adventitia, m=media, i=intima). Graph showing the media cell density based on arterial calcification (right, n = 32 CKD). **(G-H)** Graphs showing the intima (**G**) and adventitia (**H**) cell density based on arterial calcification (n = 29 CKD). **(I)** Pictures of the TUNEL assay (red) in a Ctrl and CKD artery (left). Graph showing the fold change in TUNEL-positive media cells in Ctrl (n = 8) and CKD (n = 12) arteries (P = 0.0002). **(J)** Immunohistochemical picture of cleaved-caspase 3 (cC3) in a CKD artery. This staining was done on n = 8 CKD patients. **(K-M)** Expression levels of CDKN2A (**K**), IL-6 (**L**) and TNFa (**M**) non-calcified and calcified CKD arteries (Number of non-calcified and calcified arteries, respectively: CDKN2A: n = 5, 18; IL-6: n = 4, 12; TNFa: n = 4, 12); RQ=relative quantification, A.U.=arbitrary units. **(N-O)** Expression levels of circulating IL-6 (**N**), and TNFa (**O**) in CKD patients with non-calcified and calcified arteries (Number of samples for non-calcified and calcified arteries, respectively: IL-6: n = 10, 34; TNFa: n = 10, 33). (**P**) Graph representing the frequency of progerin-positive cells in non-calcified (n = 11) and calcified (n = 39) arteries (P = 0.0357). (**Q**) The frequency of progerin-positive cells in CKD arteries correlated with the number of years for which the patients were diagnosed with the disease. Scale bars: **A** = 100μm; **F**, **I**, **J** = 50μm.Statistics: **B**-**D**, **I**, **K**-**P**: Nonparametric Mann–Whitney test with a two-tailed 95% confidence interval; **F**, **Q** (All and Cal.): Pearson correlation coefficients with a two-tailed 95% confidence interval; **G**-**H**, **Q** (Non-cal.): Spearman correlation coefficients with a two-tailed 95% confidence interval. **B**-**D**, **F**-**I**, **K**-**P**: Data presented as mean values ± SEM. *, P < 0.05; ***, P < 0.001. Source data

We investigated the impact of progerin expression on the CKD vascular phenotypes. Arteries were classified as noncalcified or calcified, with media calcification detected in 78% of patients with CKD, mainly older ones, increasing CVD risk 3.3-fold (Extended Data Fig. 3a–e). Loss of VSMCs is a common phenotype of age-related vascular decline and is one of the key features observed in HGPS arteries^27^. We analyzed the cell density of the different CKD arterial layers. Calcified arteries showed reduced media cell density, but intima and adventitia cell densities remained unchanged (Extended Data Fig. 3f–h). TUNEL assay showed increased media apoptosis in CKD arteries, though it seemed it was not caspase 3-mediated (Extended Data Fig. 3i,j). Analysis of senescence markers (CDKN2A/p16^ink4a^, interleukin (IL)-6 and TNF) showed no correlation with calcification at the RNA level (Extended Data Fig. 3k–o). Progerin expression has been associated with vascular calcification in patients and mice with HGPS^16,19^. Here, the frequency of progerin-positive cells was higher in calcified arteries and correlated with the number of years since CKD diagnosis and arterial biopsy collection, especially for patients presenting with vascular calcification (Extended Data Fig. 3p,q).

### Progerin-expressing cells proliferate in a uremic environment

**Extended Data Fig. 4:**
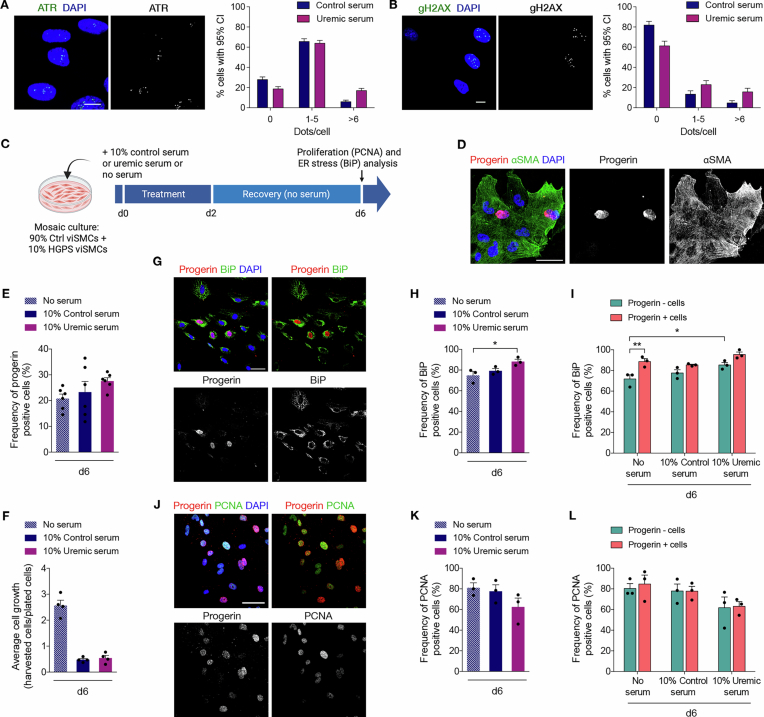
Progerin-expressing viSMCs in a mosaic setting are indistinguishable from control cells under uremic stress. **(A-B)** Persistent DNA damage in VSMCs is evident *in vitro* as observed by an increased number of foci in the cells, as measured with ATR (**A**) and gH2AX (**B**) four days post-treatment with uremic or control serum. **(C)** Schematic overview representing the experimental workflow. **(D)** Progerin (red) and αSMA (green) co-labeling showing progerin-positive viSMCs in an in vitro mosaic setting. **(E)** Graph representing the frequency of progerin-positive viSMCs 6 days after plating, following the different treatment conditions. **(F)** Graph showing the average cell growth of the mosaic cultures grown in normal conditions or treated with control/uremic serum. Cell growth was calculated as number of harvested cells/number of plated cells. **(G-I)** Immunofluorescence against progerin (red) and BiP (green) on the mosaic cultures showed an increase in ER stress after two days of treatment with 10% uremic serum (H, P = 0.0390). ER stress was increased in progerin-expressing cells vs. control cells in normal growth condition (P = 0.0092) but did not differ after uremic treatment. ER stress increased upon uremic serum in progerin-negative cells (P = 0.0396)(I). **(J-L)** Immunofluorescence against progerin (red) and PCNA (green) on the mosaic cultures showed no significant difference in proliferation after two days of treatment with 10% uremic serum (**K**). The frequency of proliferative cells did not differ between control cells and progerin-expressing cells (**L**). Scale bars: **A**-**B** = 10μm, **D**, **G**, **J** = 50μm. Statistics: Number of replicates/treatment group: **A**, **B**: n = 3, E: n = 6, F: n = 4, H-L: n = 3. **E**, **H**, **K**: One-way ANOVA with Tukey’s correction for multiple comparisons. I, L: One-way ANOVA with Tukey’s correction for multiple comparisons. **E**, **F**, **H**, **I**, **K**, **L**: Data presented as mean values ± SEM. *, P < 0.05; **, P < 0.01. Source data

The *LMNA* c.1824C>T mutation being germline in patients with HGPS, progerin is expressed in all the cells, thereupon having a detrimental effect on various tissues over time. This might make it difficult to comprehend why the mutation and progerin are recurrently present within the arterial wall of patients with CKD and at such high frequencies. To understand how progerin-expressing VSMCs function in a mosaic setting and uremic context as in CKD, we performed in vitro studies. The CKD-related uremic environment induces oxidative stress and DNA damage, which in part remains unrepaired and results in senescent cells that secrete inflammatory mediators^8^. Human VSMCs were thus treated acutely for 24 h with 10% uremic serum derived from patients with CKD (UR) or 10% control serum derived from healthy individuals (CS)^28^. Analysis 4 days post-treatment showed persistent activation of the DNA damage response (DDR), as observed by staining for γH2AX and ATR, two proteins involved in the response mechanisms to DNA double-strand breaks (Extended Data Fig. 4a,b). We then treated mosaic cultures composed of 10% of patients with HGPS and 90% control induced pluripotent stem (iPS) cell-derived smooth muscle cells (viSMCs)^29^. Mosaic cultures were subjected to 10% UR or CS or left in normal growth conditions for 2 days. Cells were analyzed 4 days post-treatment (Extended Data Fig. 4c). viSMCs were positive for αSMA and the frequency of progerin-positive viSMCs showed little variation between the different treatment groups, indicating that progerin-expressing cells remained after treatment with UR (Extended Data Fig. 4d,e). viSMCs expanded under normal growth conditions; however, their number decreased when subjected to UR or CS, which implied that cell growth was affected by treatment with human serum (Extended Data Fig. 4f). To establish whether progerin-expressing viSMCs may be more susceptible to the uremic milieu than control viSMCs, endoplasmic reticulum (ER) stress and proliferation capacity were assessed. Colocalization staining against progerin and the ER stress sensor BiP revealed an increased ER stress under uremic conditions (Extended Data Fig. 4g,h). The frequency of BiP-positive viSMCs was significantly higher in progerin-expressing cells under normal growth conditions, but increased in control viSMCs after UR treatment, suggesting that the uremic toxins present in the serum may induce ER stress (Extended Data Fig. 4i). BiP levels in progerin-expressing cells remained constant, independently from the treatment condition. ER stress was similar between control and progerin-positive cells in the uremic milieu (Extended Data Fig. 4i). Proliferation analysis via proliferating cell nuclear antigen (PCNA) showed no significant difference between the groups, but also showed that progerin-expressing cells can proliferate as well as control cells in a uremic milieu (Extended Data Fig. 4j–l). Taken together, these results suggest that progerin-expressing viSMCs are similarly affected by the uremic environment as control cells in a mosaic context, retaining a proliferative capacity.

### Indication of clonal propagation of progerin-expressing cells in CKD arteries

**Fig. 3:**
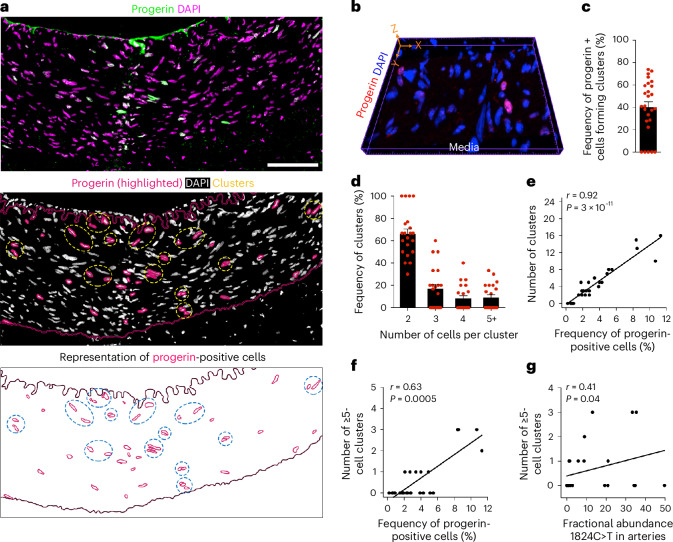
Progerin-expressing cells form VSMC clusters in CKD arteries. **a**, Immunofluorescent image showing the distribution of progerin-positive cells (green/highlighted in pink) in a CKD artery. Cell clusters are defined by yellow-/blue-dashed circles. Clusters were visualized in 21 of 32 independent CKD arteries. Scale bar, 100 μm. **b**, Spatial reconstruction based on serial artery sections showed progerin-positive cells (red) in the medial layer of a CKD artery. **c**, Graph showing the frequency of progerin-positive cells that form clusters for each CKD artery (*n* = 26). **d**, Graph showing the frequency of clusters that are formed by 2, 3, 4 or ≥5 progerin-positive cells per CKD artery (*n* = 21). **e**,**f**, Graphs showing the positive correlation between the frequency of progerin-expressing cells and the total number of clusters/sample (*n* = 26 CKD) (**e**) or the number of larger clusters/sample (*n* = 26 CKD) (**f**). **g**, Graph representing the positive correlation between the FA of the *LMNA* c.1824C>T mutation and the frequency of clusters containing ≥5 cells (*n* = 25 CKD). Statistics were Spearman correlation coefficients with a two-tailed 95% confidence interval (**e**,**f**). Data are presented as mean values ± s.e.m. (**c**,**d**). Source data

**Extended Data Fig. 5:**
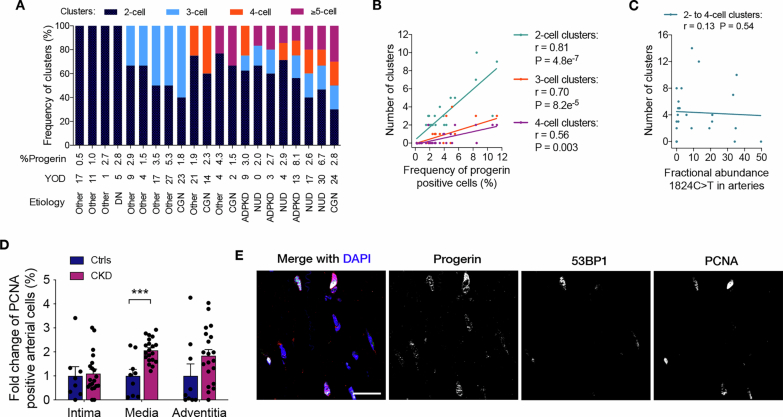
Progerin-positive cells mostly form small clusters in CKD arteries. **(A)** Graph showing the frequency of 2, 3, 4 or ≥5-cell clusters per CKD artery analyzed, and the corresponding frequency of progerin-positive cells, years of disease (YOD) and etiology. **(B)** Graph showing correlations between the frequency of progerin-positive cells and the number of 2, 3 or 4-cell clusters (n = 26 CKD). **(C)** Graph showing the positive correlation between the fractional abundance of the mutation and the frequency of clusters with 2 to 4 cells (n = 25 CKD). (**D**) Graph showing the arterial layer distribution of PCNA-positive cells in control (n = 9) and CKD (n = 20) arteries (P = 0.0035). **(E**) Colocalization staining of progerin (red), 53BP1 (green) and PCNA (white) in a CKD artery. Scale bar: **E** = 25μm. Statistics: **B**, **C**: Spearman correlation coefficients with a two-tailed 95% confidence interval. D: Multiple Mann–Whitney tests with Holm-Sidak’s correction for multiple comparisons. **D**: Data presented as mean values ± SEM. ***, P < 0.001. Source data

We next evaluated the capacity of progerin-expressing cells to clonally propagate within CKD arteries. Clusters of progerin-positive cells were found in 80.8% of patient arteries (Figs. 1b and 3a,b and Supplementary Video 1). A characterization of these clusters was performed on a subset of CKD arteries. Multiple clusters were observed within a single CKD artery section (single-plane analysis), ranging from small groups of 2–4 neighboring cells to larger clusters of ≥5 cells (Fig. 3a and Extended Data Fig. 5a). On average, 40.1% of progerin-expressing cells in a CKD artery formed clusters (Fig. 3c), with 9% of clusters containing ≥5 cells (Fig. 3d). To determine whether this distribution resulted from random independent events, we calculated the theoretical probability of neighboring progerin-positive cells^7^. Considering the highest average frequency of progerin cells in a patient with CKD (8.1%), the probability of finding two, three or four adjacent progerin cells were of 0.66%, 0.053% and 0.0043%, respectively. Together, our observations and these low probabilities suggest that the distribution of progerin-expressing cells is less likely to be random. Moreover, the frequency of progerin-positive cells correlated with both the total number of clusters and the number of larger clusters (Fig. 3e,f and Extended Data Fig. 5b). Additionally, larger clusters correlated with the allele frequency of the somatic *LMNA* c.1824C>T mutation in CKD arteries, a pattern not observed for smaller clusters (Fig. 3g and Extended Data Fig. 5c).

**Fig. 4:**
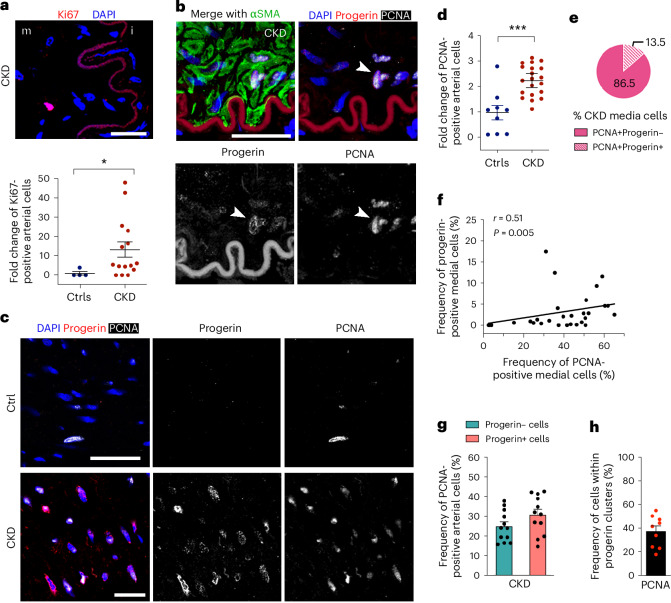
Progerin-expressing cells are positive for markers of proliferation in CKD arteries. **a**, Analysis of proliferating cells in CKD (*n* = 15) and control (*n* = 4) arteries, as detected by staining for Ki67 (red) (*P* = 0.0279). **b**, Immunostaining against progerin (red), PCNA (white) and αSMA (green) showing a cluster of proliferative progerin-positive VSMCs in a CKD artery. White arrows point to that cluster. This staining was performed on three independent CKD arteries. **c**, Immunostaining against progerin (red) and PCNA (white) showing a cluster of progerin-expressing cells in CKD and control arteries. **d**, Graph representing the fold change of PCNA-positive cells in control (*n* = 9) versus CKD (*n* = 20) arteries (*P* = 0.0006). **e**, Circle plot showing the distribution of progerin-positive cells among the PCNA^+^53BP1^−^ cells in the media layer of CKD arteries (s.d. ±12). **f**, Graph showing the correlation between the frequency of PCNA-positive cells and the frequency of progerin-positive cells. **g**, Graph representing the frequency of PCNA-positive cells present in progerin-negative and progerin-positive cells in CKD arteries (*n* = 12). **h**, Graph showing the frequency of PCNA only positive cells in progerin clusters (*n* = 9 CKD). Scale bars, 10 μm (**a**), 25 μm (**b**) and 50 μm (**c**). Statistics used were a nonparametric Mann–Whitney *U*-test with a two-tailed 95% confidence interval (**a**,**d**,**g**) and Spearman correlation coefficients with a two-tailed 95% confidence interval (**f**). Data are presented as mean ± s.e.m. (**a**,**d**,**g**,**h**). *P < 0.05; ***P < 0.001. Source data

We next assessed the proliferation status in CKD arteries. Ki67 staining was 13-fold higher in CKD arteries compared to controls (Fig. 4a). This indicated increased vascular regeneration in a tissue normally characterized by low regeneration unless damaged. Progerin and PCNA colocalization staining further revealed double-positive cells marked by αSMA expression, occasionally forming clusters indicative of clonal proliferation (Fig. 4b,c). Consistent with increased Ki67, PCNA-positive cells were 2.3-fold higher in the CKD arterial media (Fig. 4c,d and Extended Data Fig. 5d).

As PCNA can also accumulate at DNA damage sites^30^, we co-labeled progerin, PCNA and 53BP1 (a DNA damage sensor and mediator) to assess progerin expression within PCNA-positive but 53BP1-negative cells (Extended Data Fig. 5e). This showed that 13.5% of the PCNA^+^53BP1^−^ cells were also progerin-positive (Fig. 4e). The frequency of progerin-expressing cells significantly correlated with PCNA positivity (Fig. 4f); however, the frequency of PCNA^+^53BP1^−^ cells remained similar between progerin-positive and negative cells (Fig. 4g). Within progerin cell clusters, about 37.7% of the cells were PCNA^+^53BP1^−^ (Fig. 4h). Altogether, this suggested that progerin-expressing cells may clonally propagate in CKD arteries.

### Progerin-expressing VSMCs have the capacity to clonally propagate in vivo

**Fig. 5:**
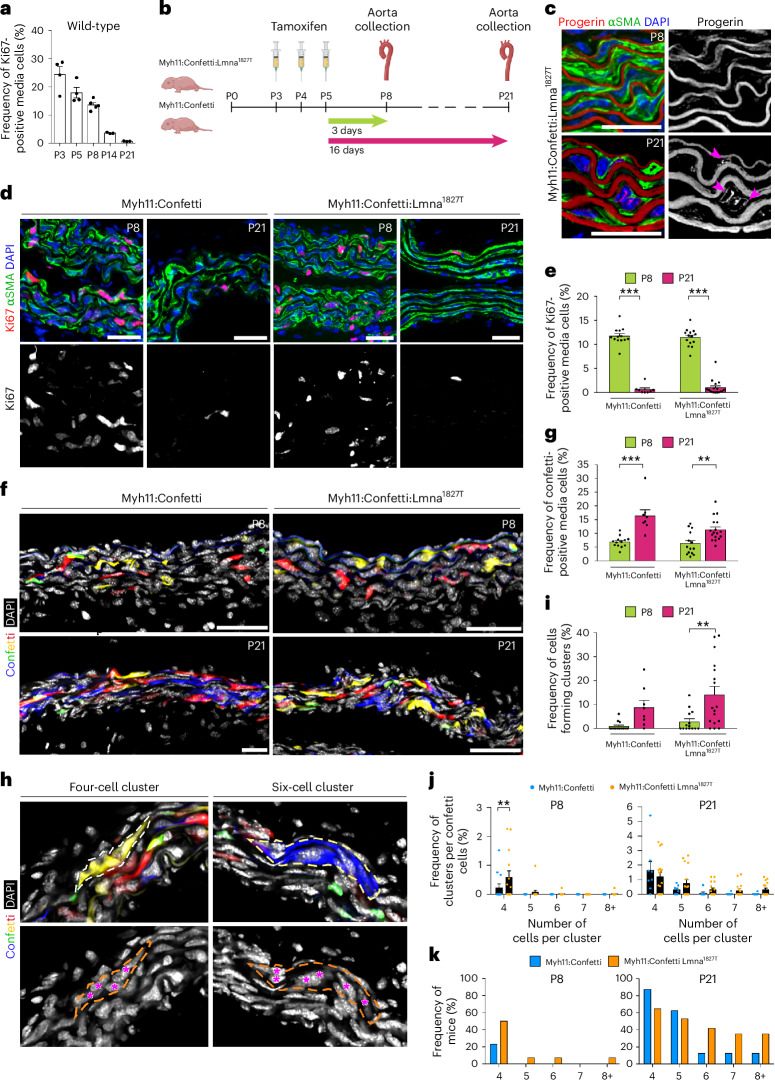
Progerin-expressing cells proliferate in the postnatal arterial wall and form clusters in vivo. **a**, Graph showing the frequency of Ki67-positive VSMCs in mice aortas (*n* = 3–5) at five different ages: P3, P5, P8, P14 and P21. **b**, Myh11:Confetti:Lmna^1827T^ and Myh11:Confetti mice were used to induce progerin and confetti reporter expression in a fraction of VSMCs. Mice underwent three consecutive tamoxifen injections starting from P3. Aortas were collected 3 and 16 days after the last dose of tamoxifen. Cartoon partly created using BioRender. **c**, Immunostaining against progerin (red) and αSMA (green) showing progerin-positive VSMCs in mosaic setting. Pink arrows point to those cells. No progerin-positive cells were detected at P8 in agreement with that progerin needs time to accumulate to be detected by the antibody. This experiment was carried out in 14 and 17 Myh11:Confetti:Lmna^1827T^ at P8 and P21, respectively. **d**, Immunostaining for Ki67 (red) and αSMA (green) at P8 and P21. **e**, Graph representing the frequency of Ki67-positive cells in Myh11:Confetti:Lmna^1827T^ and Myh11:Confetti (*P* < 1 × 10^−^^15^ for Myh11:Confetti P8 versus P21 and for Myh11:Confetti:Lmna^1827T^ P8 versus P21). **f**, Confocal pictures of confetti in mice aortas at P8 and P21. The four colors represent different confetti fluorophores: CFP (blue), GFP (green), YFP (yellow), RFP (red). **g**, Graph showing the frequency of confetti-positive VSMCs in Myh11:Confetti:Lmna^1827T^ and Myh11:Confetti mice (P8 versus P21 Myh11:Confetti, *P* = 3.4 × 10^−^^5^; P8 versus P21 Myh11:Confetti:Lmna^1827T^, *P* = 0.008). **h**, Pictures of four-cell and six-cell clusters. **i**, Graph showing the frequency of cell forming clusters in Myh11:Confetti and Myh11:Confetti:Lmna^1827T^ mice (Myh11:Confetti:Lmna^1827T^ P8 versus P21: *P* = 0.0055). **j**, Graph showing the frequency of clusters formed by 4, 5, 6, 7 or ≥8 single-color-positive cells per mice (four-cell cluster, P8 Myh11:Confetti versus Myh11:Confetti:Lmna^1827T^: *P* = 0.0045). **k**, Graph representing the frequency of mice with specific cluster sizes. Scale bars, 25 μm (**c**,**d**,**f**). Statistics were conducted using two-way ANOVA with Tukey’s correction for multiple comparisons (**e**,**g**,**i**) and an unpaired *t*-test with Holm–Sidak’s correction for multiple comparisons (**j**). Number of mice: Myh11:Confetti P8 = 13, P21 = 8; Myh11:Confetti:Lmna^1827T^ P8 = 14, P21 = 17 (**e**,**g**,**i**,**j**). Data are presented as mean ± s.e.m. (**a**,**e**,**g**,**i**,**j**). **P < 0.01; ***P < 0.001. Source data

**Extended Data Fig. 6:**
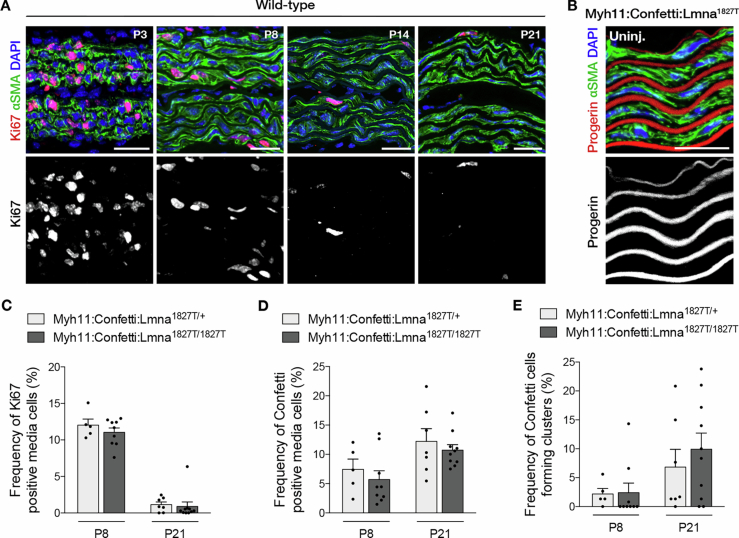
Accumulation of progerin does not interfere with the clonal propagation capacity of progerin-expressing cells. **(A)** Colocalization pictures of Ki67 (red) and αSMA (green) in aortas of wild-type mice at P3, P8, P14 and P21. **(B)** Colocalization pictures of Progerin (red) and αSMA (green) in aortas of a 5-week-old uninjected Myh11:Confetti:Lmna^1827T^ mouse. **(C)** Graph comparing the frequency of Ki67-positive cells in the media of Myh11:Confetti:Lmna^1827T/+^ and Myh11:Confetti:Lmna^1827T/1827T^ mice at P8 and P21. **(D)** Graph showing the frequency of confetti-positive cells in the media of Myh11:Confetti:Lmna^1827T/+^ and Myh11:Confetti:Lmna^1827T/1827T^ mice at P8 and P21. **(E)** Graph showing the frequency of confetti cells forming clusters in the media of Myh11:Confetti:Lmna^1827T/+^ and Myh11:Confetti:Lmna^1827T/1827T^ mice at P8 and P21. Scale bars: **A**, **B** = 25μm. Statistics: **C**-**E**: Number of mice: Myh11:Confetti:Lmna^1827T/+^ P8: n = 5, P21: n = 7; Myh11:Confetti:Lmna^1827T/1827T^ P8: n = 9, P21: n = 10. Two-way ANOVA with Tukey’s correction for multiple comparisons. Data presented as mean values ± SEM. Source data

VSMC proliferation occurs naturally during early postnatal growth. At postnatal day 8 (P8), 13.8% of the VSMCs of wild-type mice show expression of Ki67. But few VSMCs remain proliferative by P21 (Fig. 5a and Extended Data Fig. 6a). To study the clonal propagation potential of progerin-expressing cells, we crossed Myh11-CreER^T2^ mice with knock-in Lmna^LCS^ mice and R26R-Confetti mice to generate a mouse model carrying the murine equivalent to the human HGPS mutation, *Lmna* 1827C>T, and the confetti reporter specifically in VSMCs^31–33^. These mice are referred to as Myh11:Confetti:Lmna^1827T^. Homozygous and heterozygous mutants allowed for varying progerin accumulation, while Myh11:Confetti mice served as controls. To take advantage of early postnatal proliferation, mice received three consecutive injections of tamoxifen from P3 to induce recombination in a small VSMC fraction. Clonal propagation capacity of recombined VSMCs was then analyzed comparing P8 and P21 aortas (Fig. 5b).

Progerin expression was undetectable in P8 Myh11:Confetti:Lmna^1827T^ but was visible in VSMCs by P21, confirming successful recombination (Fig. 5c). Uninjected 5-week-old Myh11:Confetti:Lmna^1827T^ mice showed minimal arterial progerin (<0.5%) (Extended Data Fig. 6b). The frequencies of Ki67-positive VSMCs at P8 and P21 were similar between Myh11:Confetti and Myh11:Confetti:Lmna^1827T^ mice, indicating that mosaic progerin expression did not impact overall cell proliferation (Fig. 5d,e). Confetti recombination frequencies at P8 averaged 6.9% in Myh11:Confetti and 6.3% in Myh11:Confetti:Lmna^1827T^. While confetti-positive cells increased in P21 versus P8 aortas for both groups, progerin levels had no impact on the frequencies of Ki67 or confetti-positive cells (Fig. 5f,g and Extended Data Fig. 6c,d).

We then investigated the capacity of progerin-expressing VSMCs to form clusters by lineage-tracing. In the aortas of P21 Myh11:Confetti:Lmna^1827T^ mice, an increased number of progerin-expressing VSMCs forming clusters was observed compared to P8 mice (Fig. 5h,i). A similar trend was observed in Myh11:Confetti aortas (Fig. 5h,i). Cluster formation was unaffected by progerin levels (Extended Data Fig. 6e), but progerin-expressing VSMCs exhibited an increased tendency to form larger clusters than non-progerin cells (Fig. 5j). At P8, four-cell clusters were significantly more frequent in Myh11:Confetti:Lmna^1827T^ versus Myh11:Confetti (Fig. 5j,k). By P21, a similar trend was observed for larger clusters (≥six-cell clusters) (Fig. 5j,k). This suggests an advantage of progerin cells to form larger clones than non-progerin cells. Taken together, these data show that cells carrying the HGPS mutation in a mosaic setting can clonally propagate during proliferative events such as growth and are not outcompeted by nonmutant cells.

### Progerin contributes to ER stress, DNA damage and senescence in CKD arteries

**Fig. 6:**
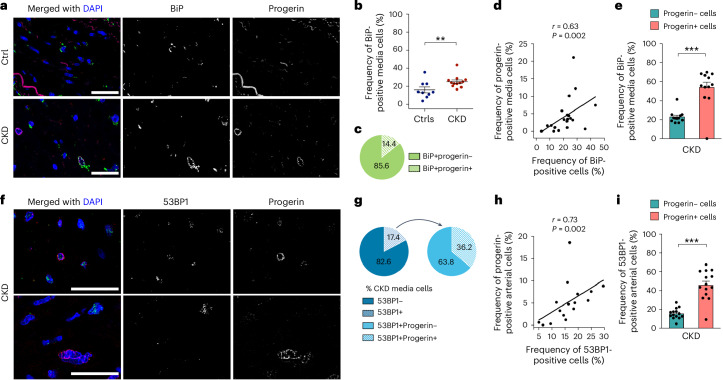
Progerin-expressing cells show molecular changes indicative of ER stress and DNA damage in CKD arteries. **a**, Colocalization staining of BiP (green) and progerin (red) in CKD arteries. **b**, Graph displaying the frequency of BiP-positive cells in the media of Ctrls (*n* = 9) versus patients with CKD (*n* = 12, *P* = 0.0073) **c**, Circle plot illustrating the distribution of progerin-expressing cells among the BiP-positive cells in the media of CKD arteries (s.d. ±11). **d**, Graph showing the correlation between the frequency of BiP-positive cells and the frequency of progerin-positive cells (*n* = 9 Ctrls and 12 CKD). **e**, Graph representing the frequency of BiP-positive cells present in progerin-negative and progerin-positive cells of CKD arteries (*n* = 12, *P* = 0.0002). **f**, Colocalization staining of 53BP1 (green) and progerin (red) in CKD arteries. This staining was conducted on 16 independent CKD arteries. **g**, Circle plots illustrating the distribution of 53BP1-positive cells in the medial layer of CKD arteries (s.d. ±1.7), as well as the overall fraction of progerin and 53BP1-positive cells. **h**, Graph showing the correlation between the frequency of 53BP1-positive cells and the frequency of progerin-positive cells (*n* = 16 CKD). **i**, Graph representing the frequency of 53BP1-positive cells present in progerin-negative and progerin-positive cells of CKD arteries (*n* = 14, *P* = 1.8 × 10^−^^5^). Scale bars, 25 μm (**a**,**f**). Statistics were nonparametric Mann–Whitney *U*-test with a two-tailed 95% confidence interval (**b**,**e**,**i**) and Spearman correlation coefficients with a two-tailed 95% confidence interval (**d**,**h**). Data are presented as mean ± s.e.m. (**b**,**e**,**i**). **P < 0.01; ***P < 0.001. Source data

ER stress has been linked to vascular calcification in CKD^34^, and progerin-induced ER stress in VSMCs accelerates atherosclerosis in HGPS mice^35^. We performed immunofluorescence staining for progerin and BiP in CKD and control arteries (Fig. 6a). BiP-positive media cells were 1.6-fold higher in CKD arteries (Fig. 6b), with 14.4% of BiP-positive cells also expressing progerin (Fig. 6c). In addition, a correlation between ER stress and progerin-positive cells was observed in CKD and control arteries (Fig. 6d). Analysis of the progerin-positive versus negative cell population in each CKD artery revealed that progerin expression was associated with a 2.3-fold increase in ER stress (Fig. 6e).

**Extended Data Fig. 7:**
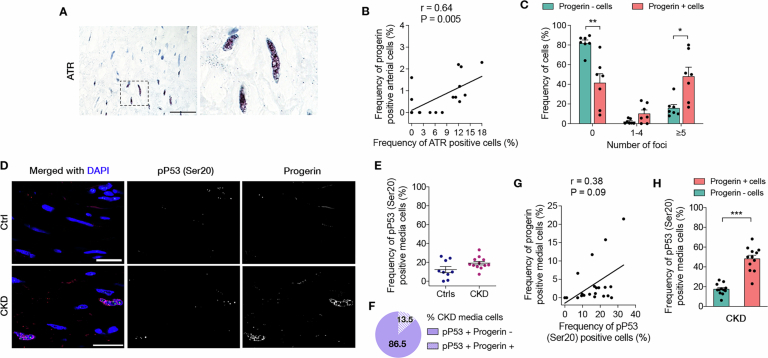
Molecular defects associate with progerin expression in CKD arteries. **(A)** Immunohistochemical staining for ATR in a CKD artery. **(B)** Graph showing the correlation between ATR accumulation and the frequency of progerin-positive cells in CKD (n = 18). **(C)** Graph representing the number of 53BP1 foci (no foci: P = 0.0035; ≥5 foci: P = 0.0139) present in progerin-negative and progerin-positive cells of CKD arteries (n = 7). (**D**) Colocalization staining of pP53 (green) and progerin (red) in arteries from CKD patients. **(E)** Graph displaying the frequency of pP53-positive cells in the media of Ctrls (n = 9) vs. CKD patients (n = 12) (P = 0.1285). **(F)** Circle plot illustrating the distribution of progerin-expressing cells among the pP53-positive cells in the media layer of CKD arteries (SD = ±12.4). **(G)** Graph showing the correlation between the frequency of pP53-positive cells and the frequency of progerin-positive cells (n = 9 Ctrls and 12 CKD). **(H)** Graph representing the frequency of pP53-positive cells present in progerin-negative and progerin-positive cells of CKD arteries (n = 12, P = 3e^-6^). Scale bars: **A** = 50μm; D = 25μm. Statistics: **B**, **G**: Spearman correlation coefficients with a two-tailed 95% confidence interval; C: Multiple Mann–Whitney test with Holm-Sidak’s correction for multiple comparisons; **E**, **H**: Nonparametric Mann–Whitney test with a two-tailed 95% confidence interval. **C**, **E**, **H**: Data presented as mean values ± SEM. *, P < 0.05; **, P < 0.01; ***, P < 0.001. Source data

DNA damage and senescence are thought to play a role in early vascular aging in CKD^36^. Persistent DDR activation is a known downstream effect of progerin expression^37^. In agreement, the frequency of ATR-positive cells correlated with that of progerin-positive cells (Extended Data Fig. 7a,b). Co-labeling of progerin and 53BP1 showed that 12.9% of CKD arterial cells were 53BP1-positive, with one quarter also positive for progerin (Fig. 6f,g). DDR activation correlated with progerin expression in CKD arteries (Fig. 6h). On average, 56.4% of progerin-positive cells had an activated DDR. In comparison, 10.1% of the progerin-negative cells were 53BP1 positive. This indicates that progerin expression may cause DDR activation, and that in the context of CKD, progerin expression is associated with 2.8-fold increased rates of DNA damage accumulation (Fig. 6i). Additionally, the frequency of cells with a high number of foci (≥5) was greater in progerin-positive than progerin-negative cells, suggesting that such cells accumulate more DNA damage (Extended Data Fig. 7c).

**Fig. 7:**
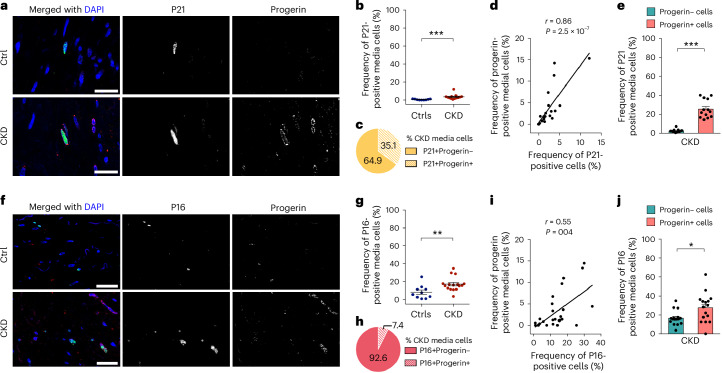
Progerin-expressing cells show molecular changes indicative of senescence in CKD arteries. **a**, Colocalization staining of P21 (green) and progerin (red) in arteries from patients with CKD. **b**, Graph displaying the frequency of P21-positive cells in the arteries of Ctrls (*n* = 9) versus patients with CKD (*n* = 13) (*P* = 4.8 × 10^−^^5^). **c**, Circle plot illustrating the distribution of progerin-expressing cells among the P21-positive cells in the media layer of CKD arteries (s.d. ±17). **d**, Graph showing the correlation between the frequency of P21-positive cells and the frequency of progerin-positive cells (*n* = 9 Ctrls and 13 CKD). **e**, Graph representing the frequency of P21-positive cells present in progerin-negative and progerin-positive cells of CKD arteries (*n* = 13) (*P* = 1.9 × 10^−^^7^). **f**, Colocalization staining of P16 (green) and progerin (red) in arteries from patients with CKD. **g**, Graph displaying the frequency of P16-positive cells in the arteries of Ctrls (*n* = 10) versus patients with CKD (*n* = 16) (*P* = 0.0035). **h**, Circle plot illustrating the distribution of progerin-expressing cells among the P16-positive cells in the media layer of CKD arteries (s.d. ±7.5). **i**, Graph showing the correlation between the frequency of P16-positive cells and the frequency of progerin-positive cells (*n* = 10 Ctrls and 16 CKD). **j**, Graph representing the frequency of P16-positive cells present in progerin-negative and progerin-positive cells of CKD arteries (*n* = 16) (*P* = 0.0194). Scale bars, 25 μm (**a**,**f**). Statistics were nonparametric Mann–Whitney *U*-test with a two-tailed 95% confidence interval (**b**,**e**,**g**,**j**) and Spearman correlation coefficients with a two-tailed 95% confidence interval (**d**,**i**). Data are presented as mean ± s.e.m. (**b**,**e**,**g**,**j**). *P < 0.05; **P < 0.01; ***P < 0.001. Source data

Earlier in vitro studies showed that DDR inhibits the growth of progerin-expressing VSMCs, and that senescence occurs in HGPS vascular cells^38,39^. We assessed the senescence phenotype in CKD and control arteries. Markers such as P21, P16 and phosphorylated P53 at serine 20 (pP53) were co-stained with progerin (Fig. 7a,f and Extended Data Fig. 7d). P21, P16 but not pP53 were more frequent in CKD arteries compared to controls (Fig. 7b,g and Extended Data Fig. 7e). In CKD, progerin was present in 35.1%, 7.4% and 13.5% of the P21, P16 and pP53-positive cells, respectively (Fig. 7c,h and Extended Data Fig. 7f). In addition, positive correlations were observed between senescence markers and progerin-expressing cells (Fig. 7d,i and Extended Data Fig. 7g). All markers were more frequent in progerin-positive cells, suggesting that cells have an increased risk of becoming senescent when expressing progerin (Fig. 7e,j and Extended Data Fig. 7h). Taken together, these results indicate that progerin-expressing cells contribute to the molecular defects associated with early vascular aging in CKD arteries.

### Progerin-expressing VSMCs induce early vascular aging phenotypes in mice

**Fig. 8:**
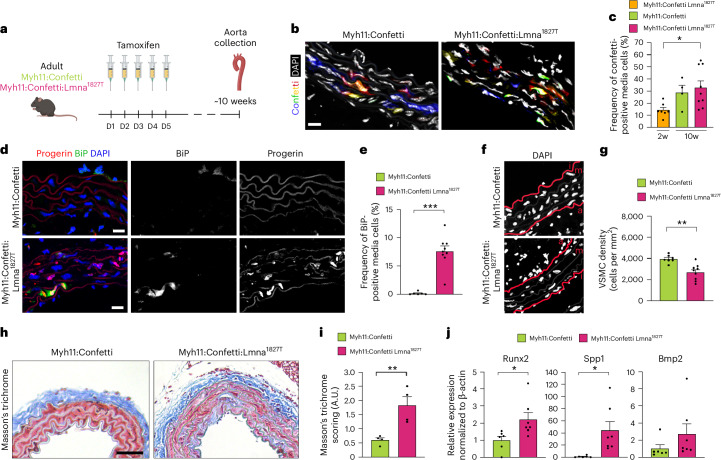
Mosaic expression of the Lmna 1827C>T mutation results in early vascular aging phenotypes. **a**, Adult Myh11:Confetti and Myh11:Confetti:Lmna^1287T^ mice were injected with tamoxifen over five consecutive days to induce mosaic progerin and confetti expression in VSMCs. Aortas were collected and analyzed about 10 weeks after the last tamoxifen injection. Cartoon partly created using BioRender. **b**, Confocal picture of confetti in the aortas of Myh11:Confetti and Myh11:Confetti:Lmna^1287T^ mice. **c**, Graph illustrating the frequency of confetti-positive cells in Myh11:Confetti and Myh11:Confetti:Lmna^1287T^ mice 2 and 10 weeks post-induction of recombination (*n* = 7 Myh11:Confetti:Lmna^1827T^ 2 weeks post-induction, *n* = 4 Myh11:Confetti and *n* = 9 Myh11:Confetti:Lmna^1827T^ 10 weeks post-induction; *P* = 0.0262). **d**, Colocalization staining of BiP (green) and progerin (red) in Myh11:Confetti and Myh11:Confetti:Lmna^1287T^ arteries. **e**, Quantification graph of BiP-positive medial cells in Myh11:Confetti (*n* = 6) versus Myh11:Confetti:Lmna^1287T^ (*n* = 9) mice (*P* = 2.1 × 10^−^^5^). **f**, Confocal picture showing reduced VSMC density. VSMC density was quantified by co-staining with αSMA (not shown) and 4,6-diamidino-2-phenylindole (DAPI) (white); a, adventitia; m, media; i, intima. **g**, Graph showing reduced vascular smooth muscle cell density in Myh11:Confetti/wild-type (*n* = 6) versus Myh11:Confetti:Lmna^1287T^ (*n* = 9) mice (*P* = 0.0025). **h**, Picture of Myh11:Confetti versus Myh11:Confetti:Lmna^1287T^ arteries stained with Masson’s trichrome. **i**, Graph showing arbitrary assessment of medial fibrosis in Myh11:Confetti/wild-type (*n* = 4) versus Myh11:Confetti:Lmna^1287T^ (*n* = 4) arteries (*P* = 0.0094). **j**, Gene expression profiling of osteogenic markers using ddPCR in Myh11:Confetti (*n* = 6) versus Myh11:Confetti:Lmna^1287T^ (*n* = 7) aortas: Runx2 (left, *P* = 0.0281); Spp1 (middle, *P* = 0.0175) and Bmp2 (right). Values are normalized to β-actin gene expression. Scale bars, 10 μm (**b**,**d**,**f**) and 50 μm (**h**). Statistics were conducted by one-way ANOVA with Tukey’s correction for multiple comparisons (**c**) and an unpaired *t*-test with a two-tailed 95% confidence interval (**e**,**g**,**i**,**j**). Data are presented as mean ± s.e.m. (**c**,**e**,**g**,**i**,**j**). *P < 0.05; **P < 0.01; ***P < 0.001. Source data

**Extended Data Fig. 8:**
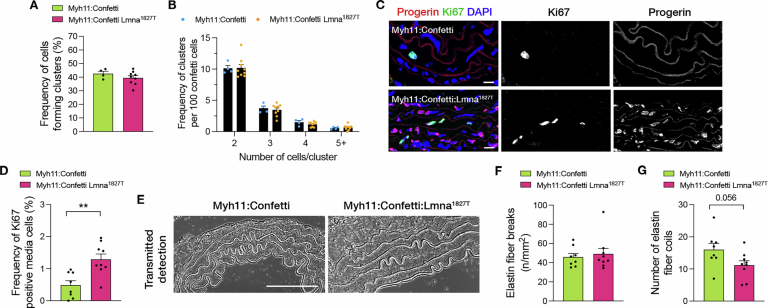
Increased Ki67 and fiber linearization upon mosaic progerin expression in mice VSMCs. **(A)** Graph showing the frequency of confetti-positive cells engaged in cluster formation in Myh11:Confetti (n = 4) and Myh11:Confetti:Lmna^1827T^ (n = 9) aortas. **(B)** Graph showing the cluster distribution per 100 confetti-positive cells in Myh11:Confetti (n = 4) and Myh11:Confetti:Lmna^1827T^ (n = 9) mice. **(C)** Co-labeling pictures of progerin (red) and Ki67 (green) in Myh11:Confetti and Myh11:Confetti:Lmna^1827T^ arteries. **(D)** Graph showing the frequency of Ki67-positive media cells in Myh11:Confetti/WT (n = 8) and Myh11:Confetti:Lmna^1827T^ (n = 9) mice (P = 0.0022). **(E)** Transmitted detection images of elastin fiber linearization and breaks. **(F-G)** Elastin fiber analyses showing the number of breaks per section (**F**) and the number of coils per section (**G**) in Myh11:Confetti/WT (n = 8) and Myh11:Confetti:Lmna^1827T^ (n = 9) mice. Scale bars: C = 10μm; **E** = 50μm. Statistics: **A**, **D**, **F**, G: Unpaired t-test with a two-tailed 95% confidence interval; B: Multiple unpaired t-test with Holm-Sidak’s correction for multiple comparisons. **A**, **B**, **D**, **F**, **G**: Data presented as mean values ± SEM. **, P < 0.01. Source data

Last, we investigated the dynamics of progerin-expressing cells with time in the vascular wall and their contribution to early vascular aging phenotypes commonly seen in CKD in vivo. Adult Myh11:Confetti and Myh11:Confetti:Lmna^1827T^ mice received five consecutive injections of tamoxifen to induce progerin and confetti expression in a fraction of VSMCs. Aortas were then collected approximately 10 weeks post-injection for analysis (Fig. 8a). Two weeks after induction, confetti expression was observed in 14.4% of VSMCs and no significant difference was observed between Myh11:Confetti and Myh11:Confetti:Lmna^1827T^ mice 10 weeks post-induction, supporting that progerin-expressing cells were not outcompeted by nonprogerin cells (Fig. 8b,c). Cluster formation and size were similar between the two groups of mice (Extended Data Fig. 8a,b). As in the arteries of patients with CKD, co-labeling for progerin and BiP showed significantly higher ER stress in Myh11:Confetti:Lmna^1827T^ mice (Fig. 8d,e).

We then assessed the impact of progerin-expressing cells on the vascular wall. Ki67 immunofluorescence revealed increased VSMC proliferation in Myh11:Confetti:Lmna^1827T^ aortas compared to controls (Extended Data Fig. 8c,d), hinting vascular regeneration in response to tissue damage. These aortas also exhibited VSMC loss (Fig. 8f,g) and increased medial fibrosis, characterized by higher collagen deposition (Fig. 8h,i). Impairment of elastin fiber organization could contribute to vascular stiffness, and while the number of elastin fiber breaks remained unchanged, we observed a trend toward more linearized elastin fibers in Myh11:Confetti:Lmna^1827T^ aortas (Extended Data Fig. 8e–g). To investigate whether progerin induces vascular calcification, we analyzed the expression of genes involved in VSMC osteogenic differentiation, finding significant upregulation in two of three markers in Myh11:Confetti:Lmna^1827T^ *vs*. Myh11:Confetti mice (Fig. 8j). These findings suggest that mosaic progerin expression in VSMCs can promote vascular calcification by inducing osteogenic differentiation. Altogether, our results show that progerin-expressing cells are not outcompeted by healthier nonprogerin cells in the mouse arterial wall and can induce mild pathological phenotypes of early vascular aging.

## Discussion

The presence and contribution of somatic mutations to the early vascular aging phenotype seen in patients with CKD remains poorly understood. Here, we identified the somatic *LMNA* c.1824C>T mutation and progerin in CKD vascular cells. To our knowledge, no previous studies have reported the existence of the germline HGPS mutation as a somatic mutation in non-HGPS individuals. While progerin has been detected in non-HGPS tissues before, the relevance of its expression and its possible contribution to aging-related defects has been less clear. We demonstrate that mosaic progerin expression contributes to the local ER stress, DNA damage and senescence phenotypes associated with early vascular aging in CKD and results in pathological vascular aging phenotypes in vivo.

The progerin-expressing VSMCs detected here were ~100–1,000 times more frequent than previously reported in non-HGPS coronary arteries^19^. This is likely due to chronic exposure to uremic toxins over an extensive time, which induces the regeneration of only a subset of VSMCs. Earlier data from lineage-tracing in mice have shown that only a limited amount of VSMCs proliferates in response to tissue damage, expanding as clones during the regeneration process of the arterial wall^7^.

Notably, the frequency of progerin-expressing cells was lower than expected from the allele frequency of the *LMNA* c.1824C>T. Such contrasting results could be explained by the lower sensitivity of protein detection compared to DNA rare event detection, low expression of progerin in certain cell types^40,41^ and/or allele-specific expression of the *LMNA* locus^42^. It could also be explained by partial activation of the cryptic splice site, as the presence of the c.1824C>T variant is not excluding full length lamin A splicing from that allele^14^.

Although one study showed that progerin expression in the vascular wall increases with aging^19^, such observation was not visible in the present CKD cohort. That study included individuals aged from 1 month to 97 years, whereas our patients with CKD were aged 20–69 years. There was, however, a correlation between the frequency of progerin-positive cells and the number of years since CKD diagnosis. Our CKD population is quite heterogenous as some patients were undiagnosed for many years, and it is difficult to know exactly how long they have had kidney damage and their arteries have been exposed to mutagens such as uremic toxins. In addition, patients have different disease etiology, and some have been on dialysis before arterial sampling. This heterogeneity as well as the short age range of our sample collection might be limiting this correlation.

The recurrence of the *LMNA* c.1824C>T mutation among different CKD individuals is surprising. Especially as neither the presence of the mutation nor progerin expression showed association with any specific etiology of CKD. This observation suggests that the occurrence of the mutation is secondary to the renal impairment and instead represents somatic evolution of cells to survive the uremic environment; however, we cannot exclude that prolonged exposure to uremic toxins results in the induction of progerin-expressing VSMCs. Mutational hotspots can be found in the genome, including at CpG-rich loci^43^. Indeed, the increased mutability of CpG dinucleotides was demonstrated in various disease-associated genes, such as *DMD*, *RB1* and *LMNA*^14,44,45^. Previous work estimated that the mutational rate at CpG loci was five times higher than the base mutational rate^46^. In addition, GG nucleotides surround the *LMNA* 1824C allele, forming the GGCGG motif, which is considered a hotspot for germline mutations in inherited diseases^47^. These elements suggest that this *LMNA* locus is a hotspot. Future sequencing of somatic cells will shed light on frequencies for somatic mutation hotspots in differentiated cells and tissues.

The recurrence of the mutation within multiple cells of the CKD arteries, on the other hand, could be explained by the clonal nature of the vascular tissue in disease as previously shown in mice^1,7^. Clonality in CKD arteries was supported by clusters of neighboring progerin-expressing cells. Although we cannot exclude that cells may be moving around in the medial layer or that the mutation may arise independently in these CKD arteries, we propose that in response to tissue damage, mutant VSMCs may dedifferentiate and clonally propagate^48^. Indeed, several studies have shown that progerin-expressing cells can proliferate and initially have a growth advantage^20,49,50^. This could be explained by the fact that progerin-expressing cells were shown to accumulate structural genetic variations over time^51^, which might result in less controlled cell growth. Accordingly, we showed that progerin-expressing cells can proliferate like non-progerin cells in a mosaic setting and under acute uremic conditions. Our lineage-tracing experiment in mice confirmed that mutant VSMCs could clonally expand and form larger clones than nonmutant cells; however, progerin accumulation disrupts cell functions^52^. Here, somatic progerin expression played a role in the ER stress, DNA damage and senescence phenotypes seen in patients with CKD and its mosaic expression in mice resulted in pathological early vascular aging phenotypes. We suggest that progerin accumulation in CKD VSMCs leads to persistent DNA damage and ER stress. Progerin-expressing VSMCs eventually enter senescence^53^ and finally cell death. These processes would thus contribute to (though are not solely responsible for) early vascular aging in patients with CKD.

In summary, our findings highlight that clonal occurrence of certain somatic mutations could become a risk when increased proliferation is induced by extensive tissue damage. Analyzing somatic genetic variations in age-associated diseases and studying rare conditions like HGPS are crucial as it may undercover novel disease contributors relevant to public health priorities.

## Methods

### Ethical approval

Human studies were approved by the regional committee of ethics in Stockholm and adhered to the statuses of the Declaration of Helsinki (Ethical Permits nos. 2008/1748-31/2, 2011/668-31/3, 2015/1115-31 and 244/01). All patients provided written informed consent before enrollment. Animal studies were approved by Linköping’s regional animal research ethical review board (6088-2020). All procedures were performed in accordance with the institutional guidelines and regulations.

### Human samples

Regional cohorts from the Stockholm area in Sweden were used. Consecutive adult patients with CKD stage 5 (eGFR<15 ml min^−1^) undergoing living-donor renal transplantation (LD-RTx) at the Department of Transplantation Surgery at Karolinska University Hospital were invited to participate in the study. Patients with CKD stage 5 undergoing living-donor transplantation constitute a healthier selection than patients with CKD stage 5 remaining on dialysis, with less CVD and less vascular calcification. Epigastric arteries were obtained from 50 patients with CKD stage 5 during LD-RTx. Media calcification score of epigastric arteries from patients with CKD is a good predictor of cardiovascular events and mortality, making this type of artery relevant to study CVD in the context of CKD^54^. Control arteries were obtained from 24 patients without history of CVD, who underwent surgery for inguinal hernia or laparoscopic cholecystectomy for gallstone disease. Removing a small piece of the artery during surgery is not an uncommon procedure, and no complications have been reported from any of the patients involved in this study. Femoral control arteries were collected from ten deceased individuals with history of CVD (approved by the Uppsala Ethics Board Review, no. 2014/500/1). Blood samples were obtained from 26 of the 50 patients with CKD stage 5 and 26 controls. Basic characteristics of the patients with CKD and controls are outlined in Supplementary Table 1. The causes of CKD were chronic glomerulonephritis (*n* = 14), adult polycystic kidney disease (*n* = 8), diabetes (*n* = 9), non une description (*n* = 7) and other (or unknown) renal diseases (*n* = 12). The most commonly used medications were erythropoiesis-stimulating agents (*n* = 38), active vitamin D (*n* = 43), angiotensin-converting-enzyme inhibitors and/or angiotensin receptor blockers (*n* = 29). Before RTx, 13 of the patients had been diagnosed with cerebrovascular (*n* = 2), cardiovascular (*n* = 6) and/or peripheral vascular disease (*n* = 5) (grouped as CVD). Out of the 50 patients, 20 received conservative treatment before undergoing pre-emptive LD-RTx, while 30 patients underwent dialysis treatment before LD-RTx for a median period of 0.5 years, by hemodialysis (*n* = 16), by peritoneal dialysis (*n* = 13) or both, as one patient who initially received hemodialysis, later switched to peritoneal dialysis.

### Arterial biopsies

Arterial biopsies were obtained within 20 min after skin incision at the start of surgery. One piece (1–2 cm in length) of the inferior epigastric artery was collected by sharp dissection. Samples were formalin-fixed and paraffin-embedded (FFPE; 4% formalin). Control arteries underwent the same procedure.

### Biomarker measurements

Blood samples were obtained from patients in a fasting state in the morning of the day before or on the day of surgery, and stored at −80 °C. Measurements were conducted as previously described^55^. In short, IGF-1, IL-6 and TNF were analyzed by immunometric assays on an Immulite 1000 Analyzer (Siemens Healthcare Diagnostics) according to the instructions of the manufacturer. Osteoprotegerin concentration and human FGF23 (C-terminal) were measured by ELISA (MicroVue OPG assay, Quidel Corporation; Immutopics International). Klotho was measured by human soluble α-Klotho ELISA Assay (IBL International). Analyses of high-sensitivity C-reactive protein (hsCRP), parathyroid hormone (PTH), plasma cholesterol, triglycerides, creatinine, albumin, calcium, phosphate and vitamin D were performed according to validated routine methods at the Clinical Laboratory of the Karolinska University Hospital, Stockholm, Sweden.

### Tissue immunofluorescence

FFPE arteries from patients with CKD, controls and CVD controls were cut into 5-μm sections. To reduce the high background caused by calcium deposits, sections were decalcified by incubation in EDTA 12.5% for 24 h at room temperature before staining. Before labeling, the sections were rehydrated, followed by heat-mediated antigen retrieval using either 0.5 M EDTA pH 8 in a pressure cooker, 1× Tris–EDTA, pH 9 in a water bath or 10 mM sodium citrate buffer, pH 6 in a microwave. Blocking was performed with 20% normalized goat or rabbit serum in combination with 1% BSA. After overnight incubation at 4 °C with the primary antibodies, anti-human progerin (1:150 dilution, 13A4, Enzo Life Science), anti-CD31 (1:50 dilution, ab28364, Abcam), anti-human prelamin A (1:150 dilution, sc-6214, Santa Cruz Biotechnology), anti-BiP (1:1,200, ab21685, Abcam), anti-BiP (1:200 dilution, C50B12, 3177, Cell Signaling), anti-53BP1 (1:200 dilution, ab36823, Abcam), anti-P21 Waf1/Cip1 (1:200 dilution, F-5, sc-6246, Santa Cruz Biotechnology), anti-P16 (1:1,000 dilution, ab54210, Abcam), anti-phospho-p53 (ser20) (1:150 dilution, 9287, Cell Signaling), anti-ki67 (1:150 dilution, clone MM1, Vector Laboratories), anti-PCNA (1:2,000 dilution, ab18197, Abcam) or anti-ki67 (1:500 dilution, ab15580, Abcam), samples were incubated for 45 min at room temperature with the appropriate secondary antibodies, Alexa 488-conjugated goat anti-rabbit (1:500 dilution, A11034, Invitrogen), Alexa 555-conjugated goat anti-mouse (1:150 dilution, A21422, Invitrogen) and Alexa 633-conjugated rabbit anti-goat (1:150 dilution, A21086, Invitrogen). Anti-actin α-smooth muscle-Cy3 (1:3,000 dilution, C6198, Sigma-Aldrich) was added for 1 h. Sections were counterstained with DRAQ5 or 4,6-diamidino-2-phenylindole (DAPI; 1:1,000 dilution, Thermo Fisher Scientific) for 5 min before mounting (ProLong Gold antifade reagent, Molecular Probes). Imaging was performed on a Nikon A1R coupled to a Digital Sight 10 CMOS color camera and a Nikon spinning-disk CREST v.3 coupled to a Kinetix sCMOS camera (Nikon Corporation) using a ×60 oil objective, and images were analyzed using NIS elements. Each staining was manually analyzed and quantified, except for the cell density analysis in patient arteries where automated DAPI nuclei counting was performed using Imaris (v.9.9.0). Progerin-positive cell clusters were analyzed in CKD artery sections where a minimum of two progerin-positive cells could be observed (one 5-μm-thick section per sample). Progerin-positive cell clusters were defined as two or more neighboring cells in close proximity (≤50 μm), uninterrupted by nonprogerin-expressing cells and based on convergent nuclei orientation. The theoretical distribution of progerin-positive cells was calculated as the highest average frequency of progerin-expressing cells is 8.1% in a patient with CKD. The probability of finding two-cell clusters was 0.081 × 0.081 = 0.66%; three-cell clusters was 0.081 × 0.081 × 0.081 = 0.053%; and four-cell clusters was 0.081 × 0.081 × 0.081 × 0.081 = 0.0043%.

### Western blot

Protein extraction and western blotting were performed as previously described^50^. Proteins originated from skin samples collected from mice overexpressing the human *LMNA* c.1824C>T mutation (*n* = 3) or from wild-type mice (*n* = 1)^50^. Proteins were subjected to brief sonication before western blotting. Antibodies against lamin A/C (1:200 dilution, N18, Santa Cruz Biotechnologies), progerin (1:200 dilution, 13A4, Enzo Life Science) and β-actin (1:5,000 dilution, A5441, Sigma-Aldrich) were used. The corresponding secondary antibodies were as follow: HRP-conjugated goat anti-mouse IgG (1:10,000 dilution, Jackson ImmunoResearch) and rabbit anti-goat IgG (1:10,000 dilution, Jackson ImmunoResearch).

### Tissue immunohistochemistry

Tissue immunohistochemistry was performed on 5-μm-thick sections of FFPE arteries. Samples were decalcified for up to 72 h by immersion in EDTA 12.5%, pH 7. Sections were rehydrated and stained for anti-human prelamin A (1:150 dilution, sc-6214, Santa Cruz Biotechnology), anti-cleaved-caspase 3 (1:200 dilution, 9664S, Cell Signaling Technology) and anti-ATR (1:350 dilution, phospho Thr1989, GTX128145, GeneTex). Tissue sections were subjected to heat-induced epitope retrieval by incubation in 10 mM sodium citrate buffer, pH 6. Endogenous peroxidase activity was blocked using a 2.5% hydrogen peroxide solution, followed by blocking with 3% normal goat serum for 30 min. The primary antibody was applied and incubated overnight at 4 °C. The secondary antibody, biotin-goat anti-rabbit IgG (1:500 and 1:1,600 dilution, respectively; Zymed 65-6140, Invitrogen) was then added for 30–45 min, followed by the label antibody (ABC Elite, Vector Laboratories) for 30 min. DAB chromogen (Dako Cytomation) was applied for 2 min, followed by two rinses in distilled water. Sections were counterstained with Mayers hematoxylin (Histolab) and mounted with mounting medium for light microscopy (Pertex). Imaging was performed using a Nikon E1000 coupled to a Nikon DXM1200 camera. Each staining was manually analyzed and quantified.

### Telomere length measurement

Telomere length in the DNA samples was measured by qPCR, following the method previously described by Cawthon^56^. Each sample was analyzed in triplicate using primer sets specific for telomere length and a single-copy gene amplicon 36B4 (acidic ribosomal phosphoprotein). The relative T:S ratio (repeat copy number to single-copy gene number) for each experimental sample was determined in relation to the control DNA sample. The inter-assay coefficient of variance was on average 0.32% for telomere and 0.12% for 36B4 respectively.

### DNA and RNA isolation from biopsies and FFPE arteries

Artery biopsies from patients and controls were collected in Allprotect Tissue Reagent (QIAGEN) and total RNA was isolated using either TRIzol Reagent (Ambion, Life Technologies) or RNeasy Plus Universal kit (QIAGEN). SuperScript III (Invitrogen, Life Technologies) was used for cDNA synthesis with random hexamers. Blood DNA was isolated using the QIAamp DNA Blood Maxi kit (QIAGEN) following the manufacturer’s protocol. RNA and DNA yields were measured using NanoDrop ND-1000 spectrophotometer (NanoDrop products) or the Qubit 3.0 Fluorometer (Thermo Fisher Scientific). RNA quality was evaluated on an Agilent 2100 BioAnalyzer chip (Agilent Technologies). DNA was also extracted from 5-μm-thick FFPE sections from patients with CKD and controls. When arterial sections were stained before DNA isolation, slides were incubated in PBS at 37 °C with gentle shaking for 1 h to remove the mounting media and coverslips. When unstained, sections were deparaffinized and placed at 97.5 °C in 10 mM sodium citrate buffer, pH 6 for 40 min. For DNA extraction, the QiAmp DNA extraction micro kit (QIAGEN) was used. The whole section was scraped and rinsed off the slide using a mix of ATL buffer and Proteinase K, and the isolation was performed following the manufacturer’s instructions for ‘Isolation of Genomic DNA from Laser-Microdissected Tissues’.

### Preparation of standards for ddPCR

HGPS B-lymphoblastoid cells were obtained from the Coriell Cell Repository (AG10587). The cell line was used as a heterozygous standard for rare event detection testing with ddPCR. Lymphoblasts were cultured in RPMI 1640 medium supplemented with 15% fetal bovine serum (FBS), penicillin–streptomycin and l-glutamine. Cells were incubated at 37 °C in 5% CO_2_. The viability of cells was monitored using an inverted light microscope and cells were counted using a hemocytometer. For mutations in the *DMD*, *EGFR*, *CFTR* and *LAMA2* genes, 500-bp gBlocks Gene Fragments (Integrated DNA Technology) were used as standards, following the manufacturer’s protocol.

### Rare event detection by ddPCR

Rare event detection was performed using the QX200 ddPCR system (Bio-Rad). DdPCR assays were designed for six single nucleotide variants (*LMNA* c.1824C>T; *EGFR* c.2369C>T; *DMD* c.8689C>T; *CFTR* c.1898+1G>A; *DMD* c.9771+1G>A; *LAMA2* c.3973+2T>C). PCR thermal cycling conditions were optimized for each individual assay. PCR reactions were performed according to the manufacturer´s protocol using 2× ddPCR Supermix for Probes (no dUTP, Bio-Rad), 20× ddPCR assay (FAM/HEX labeled, Bio-Rad), 5 U of *Hind*III restriction enzyme (New England BioLabs) and template DNA. Raw fluorescence data for each well were analyzed using QuantaSoft v.1.6 (Bio-Rad). The *LMNA* c.1824C>T assay was tested on B-lymphoblastoid cells from patients with HGPS, where the mutation had previously been confirmed by Sanger sequencing. The threshold delimiting positive from negative droplets was defined according to the DNA from the HGPS sample or the gBlocks Gene Fragments.

For the analysis of DNA extracted from FFPE arterial sections, all available DNA was used and run in one well. For the PBMC analysis, 30 ng of sample DNA was used per well, and each sample was run in duplicates. The data were then merged to calculate the FA of the mutant allele. In each run, DNA extracted from the tail of a transgenic mouse with the human lamin A minigene integrated in its genome was included, which served as an additional negative sample for quality control^50^.

Sample data were only included in the analysis when ≥3 positive droplets per sample were detected and ≥10,000 accepted droplets per well were obtained. Additionally, the limit of detection for the *LMNA* c.1824C>T assay was determined using serial dilutions of HGPS mouse tail DNA^50^. Dilutions were prepared with calculated DNA concentrations of 0.04 ng, 0.48 ng, 1 ng and 4.4 ng (Qubit 2.0, Invitrogen). The *LMNA* c.1824C>T assay was run on four replicates of each serial dilutions, and the DNA input from each replicate was measured. The s.d. and coefficient of variation (CV) were calculated. We determined the limit of detection based on a %CV < 20%, which corresponded to an average number of haploid genomes of 88.5 (Table 1).

**Table 1:** Limit of detection of the LMNA c.1824C>T assay

Calculated DNA concentration (ng)	Measured DNA concentration (ng)	Average haploid genomes	s.d.	%CV
0.04	0.04	11.10	0.02	48.65
0.48	0.3	88.50	0.03	8.92
1	0.68	205.00	0.08	11.38
4.4	3.00	900.20	0.05	1.76

Sample data were included in the analysis when the number of haploid genomes was ≥88.5. Out of the 50 CKD artery samples tested, 46 samples had sufficient DNA input and were above the limit of detection.

### In vitro assessment of PFA-induced allelic imbalance

B-lymphoblastoid cells were obtained from the Coriell Cell Repository (AG03506, AG03504). Cells were cultured with RPMI 1640 medium (Gibco, Thermo Fisher Scientific) containing 5% FBS and 1% penicillin–streptomycin. Mosaic cell cultures were generated by mixing HGPS patient B-lymphoblastoid cells (carrying the *LMNA* c.1824C>T variant) and wild-type B-lymphoblastoid cells to achieve a *LMNA* c.1824C>T FA of 50%, 7% and 5%. After reaching confluency, medium was removed and cells were treated for 30 min with 4% paraformaldehyde (PFA) at room temperature. After rinsing with PBS, a de-crosslinking step was performed by incubating the mosaic cell cultures at 96 °C for 10 min. DNA was isolated following the manufacturer’s protocol for ddPCR analysis (Gentra Puregene Cell kit, QIAGEN). Then, 50 ng of sample DNA was used for ddPCR, and each sample was run in duplicates. The FA was calculated from merged duplicates.

### Allele fraction analysis of long-term FFPE tissues

Aorta and heart tissues from humanized *LMNA* mice^50^ were used. The 5-μm sections were obtained from >15-year-old FFPE tissues. DNA was isolated as described above, and further treated with uracil-DNA glycosylase (UDG) (Thermo Scientific, FEREN0361) to eliminate FFPE-induced C:G to T:A transitions^57^. Enzyme activity was calibrated to obtain maximal efficiency. DNA samples were incubated with 1 U UDG at 37 °C for 60 min as described by the manufacturer’s protocol. Following UDG treatment, DNA samples were analyzed by ddPCR for the *LMNA* c.1824C>T allelic fraction, as described above.

### Calcification assessment

Von Kossa staining was performed on 5-μm-thick CKD arterial sections, imaged using Ocus40 Digital microscope scanner (Grundium). An experienced pathologist evaluated the sections for vascular calcification.

### CDKN2A gene expression

Real-time qPCR was performed in triplicate with TaqMan gene expression assays specific to CDKN2A/p16 (Hs00923894_m1) and normalized to the reference gene *HPRT1* (Hs02800695_m1) using 7500 Fast Real Time PCR (Applied Biosystems, Life Technologies) as previously described^55^. The comparative threshold cycle method (ΔΔCT) was used to quantify relative gene expression and the obtained quantification was transformed to exponential value 2^-ΔΔCT^ (ref. ^58^).

### TUNEL assay

Cell death was determined using the In Situ Cell Death Detection kit, TMR red (Roche) in accordance with the manufacturer’s protocol. The kit was used on 5-μm-thick sections of FFPE arteries from CKD and control arteries. Nuclei were counterstained with DRAQ5 (1:1,000 dilution, Thermo Fisher Scientific). Imaging was performed on a Nikon A1R and an A1+ imaging system (Nikon Corporation) using a ×60 oil objective, and images were analyzed using NIS elements. TUNEL was manually quantified.

### In vitro treatment of VSMCs with uremic serum

Human primary aortic smooth muscle cells (ATCC, PCS-100-012, LGCstandards) were grown in Vascular Cell Basal Medium (ATCC, PCS-100-030) supplemented with a Vascular Smooth Muscle Cell Growth kit (ATCC, PCS-100-042) for 24 h. Cells were separated in two groups. The first group received 10% uremic serum (collected from four patients with CKD stage 5 before dialysis, and pooled for the experiment) and the second group received 10% healthy serum (derived and pooled from four healthy individuals). After 24 h, medium was replaced by growth medium (Endothelial Basal Medium, CC-3121, supplemented with EGM-MV SingleQuots, CC-4143, Lonza). After 4 days in culture, cells were fixed and stained for markers for DNA damage, and DNA isolated (Gentra Puregene Cell kit, QIAGEN) for ddPCR analysis.

### viSMC differentiation and culture

Primary skin fibroblasts (168 CL2) were provided by the Progeria Research Foundation and converted to iPS cells by the Duke iPSC Shared Resource Facility. iPS cells with the classic, heterozygous *LMNA* 1824C>T HGPS mutation (003 CL1D) were provided by the Progeria Research Foundation. The iPS cells were maintained in feeder-free conditions on human embryonic stem cell-qualified Matrigel (BD Biosciences) in mTeSR Plus (Stem Cell Technologies). iPS cells were passaged at 80–90% confluency with 0.5 mM EDTA (Invitrogen) for maintenance culture or with Accutase (Stem Cell Technologies) and 10 µM ROCK inhibitor Y-27632 (Tocris Bioscience) for differentiation. viSMCs were differentiated using a modification of the protocol by Patsch et al. as previously described^29,59^. iPS cells were dissociated on day 0 with Accutase and re-plated on Matrigel coated plates at a density of 37,000 cells per cm^2^. On day 1, the medium was replaced with mesoderm induction medium consisting of N2B27 medium (1:1 mix of Neurobasal medium and DMEM/F12 with HEPES supplemented with N2 and B27 minus vitamin A, all Gibco) with 25 ng ml^−1^ BMP4 (PeproTech) and 8 µM CHIR99021 (Cayman Chemical). The medium was not changed for 3 days. On day 4 the medium was changed to viSMC induction medium consisting of N2B27 medium supplemented with 10 ng ml^−1^ PDGF-BB (PeproTech) and 2 ng ml^−1^ Activin A (PeproTech). The viSMC induction medium was changed daily. On day 6, cells were dissociated with Accutase and re-plated on collagen-coated plates in viSMC medium comprised of N2B27 medium supplemented with 2 ng ml^−1^ Activin A and 2 µg ml^−1^ heparin (Sigma-Aldrich) to induce a contractile smooth muscle cell phenotype. The medium was changed every other day and viSMCs were routinely cultured on collagen-coated plates and passaged at 80–90% confluency using Accutase. viSMCs were characterized by anti-actin α-smooth muscle-Cy3 (1:3,000 dilution, C6198, Sigma-Aldrich) staining. viSMCs were used between passages 3–4.

### In vitro sera treatment of mosaic viSMC cultures

Mosaic viSMC cultures were composed of 10% of patients with HGPS and 90% control cells grown on coverslips. Cultures were separated into three treatment groups in which the viSMC culture medium was supplemented either with 10% uremic serum (collected from 15 patients with CKD stage 5 before dialysis, and pooled for the experiment) in a first group or 10% control serum (pooled from 30 healthy individuals) in a second group. The third culture group was kept in normal growth conditions, without serum supplementation (control group). Cells were treated for 48 h (day 2), after which the serum-supplemented medium was replaced by viSMC culture medium. After 4 days of recovery, cells were fixed with 4% PFA and processed for immunofluorescence.

### In vitro immunostaining

VSMCs and mosaic viSMC cultures grown on coverslips were fixed with 4% PFA for 15 min and rinsed with PBS. Cells were permeabilized with 1% NP40 (Thermo Fisher Scientific). Blocking was performed with 20% normalized goat or rabbit serum in combination with 1% BSA. After overnight incubation at 4 °C with the primary antibodies, anti-phospho-histone H2AX (Ser139, clone JBW301, 05-636, Merck Millipore), anti-ATR (phospho Thr1989, GTX128145, GeneTex), anti-progerin (1:150 dilution, 13A4, Enzo Life Science), anti-BiP (1:400 dilution, ab21685, Abcam) and anti-PCNA (1:1,500 dilution, ab18197, Abcam), samples were incubated for 30 min at room temperature with the appropriate secondary antibodies. Samples were counterstained with DAPI (1:500 dilution, Thermo Fisher Scientific) for 5 min before mounting (ProLong Gold antifade reagent, Molecular Probes). Imaging was performed on a Nikon A1R and an A1+ imaging system (Nikon Corporation), and automated measurements were performed using NIS elements.

### Spatial reconstruction

The CKD FFPE artery was cut into five consecutive, 5-μm sections. Sections were processed for immunofluorescence staining against progerin as described in the previous section ‘Tissue immunofluorescence’. Imaging was performed using a Nikon A1R and an A1+ imaging system (Nikon Corporation) using a ×40 silicon oil objective. Individual images were imported and aligned in Imaris (Oxford Instruments, v.9.9.0). For spatial reconstruction, the aligned sections were displayed using the three-dimensional view function in Imaris. The animated video was created using the animation function in Imaris.

### Experimental mice

Experimental mice used in this study were males in a C57BL/6J background. Myh11-CreER^T2^ mice were ordered from The Jackson Laboratory^31^. Lmna^LCS^ (carrying the murine HGPS mutation *Lmna* c.1827C>T) and R26R-Confetti mice were a kind gift from V. Andrés and P. Katajisto, respectively. These mice were described previously^32,33^. Mice were crossed to generate Myh11:Confetti, Myh11:Confetti:Lmna^1827T/+^ and Myh11:Confeti:Lmna^1827T/1827T^. Mice were housed in ventilated cages with no more than five mice per cage in a pathogen-free animal facility at Karolinska Institutet, Campus Flemingsberg, Sweden. They were maintained in a 12-h light–dark cycle, at 20–22 °C temperature and 50–65% air humidity, with ad libitum access to water and irradiated rodent chow (Teklad Global diet containing 18% protein, 6% fat and moderate phytoestrogen, 2918, Inotiv).

### Induction of recombination in pups

Birth of pups was monitored daily. The day pups were born was defined as P0. Whole litters were injected intraperitoneally with tamoxifen (75–100 mg kg^−1^) on three consecutive days starting at P3. Pups were killed either 3 days or 16 days after the last injection occurred. Only males were analyzed as the Myh11-CreER^T2^ BAC transgene is inserted in the Y chromosome. Aortic arches and upper descending aortas were fixed in 4% PFA for 20 min, then incubated in 30% sucrose overnight at 4 °C to be later embedded in OCT compound. Lower descending aortas were fixed in 4% PFA overnight at 4 °C, dehydrated and embedded in paraffin. Immunofluorescence staining for progerin, Ki67 and αSMA was performed as described above in the ‘Tissue immunofluorescence’ section.

### Confetti analysis

For confetti analysis, 14-μm-thick cryosections were cut and stained with DAPI (1:800 dilution, Thermo Fisher Scientific) for 5 min before mounting (ProLong Gold antifade reagent, Molecular Probes). Arterial sections were imaged using a Nikon A1R and A1+ confocal laser scanning microscopy system (Nikon Corporation). The imaging setup comprised five distinct lasers (channels) and associated virtual emission filters, designed to maximize signal detection while minimizing spectral overlap. Tissues were imaged using a ×20 air objective lens. Imaging involved capturing tiled Z-stacks over nine iterations, with a 2-μm separation between adjacent slices. For the purposes of recombination rate calculations, one of the middle planes with the focus set on DAPI was selected. The analysis of cluster formation was executed using all available Z-stack data. A cell cluster was defined as at least four cells of the same confetti color, located adjacent to each other within the medial layer of an artery. Pictures were analyzed using NIS Elements Viewer and NIS Elements AR analysis software (Nikon, v.6.02.01).

### Induction of recombination in adult mice

Males Myh11:Confetti:Lmna^1827T/1827T^ mice aged between 5 and 13 weeks of age were used. Age- and sex-matched Myh11:Confetti or wild-type mice were used as controls. Mice were injected intraperitoneally with tamoxifen (100 mg kg^−1^) on five consecutive days. Aortas were collected about 10 weeks after the last injection for analysis. Aortic arches and upper descending aortas were fixed in 4% PFA for 20 min, then incubated in 30% sucrose overnight at 4 °C to be later embedded in OCT compound. Distal aortic arch and lower descending aortas were fixed in 4% PFA overnight at 4 °C, dehydrated and embedded in paraffin. Immunofluorescence staining (progerin, Ki67, αSMA and BiP) and confetti analysis were performed on both aortic arches and descending aortas as described above in the ‘Tissue immunofluorescence’ and ‘Confetti analysis’ sections. Cell clusters were defined as two or more adjacent cells of the same confetti color. VSMC density analysis was performed by automated DAPI nuclei counting using NIS-element AR (Nikon, v.6.02.01) after delimiting the medial area based on αSMA-positive cells.

### Medial fibrosis in mice

To detect medial fibrosis, Masson’s trichrome stain was performed on 4-μm-thick FFPE sections and imaged with a Leica DMLB microscope connected to a Zeiss AxioCam ICc 5 camera (Carl Zeiss) using a ×20 air objective. Each section was given an arbitrary score based on area covered with blue staining (0, negative; 1, weak positive; 2, positive and 3, strong positive). Sections were independently graded by two individuals and scores were merged.

### RNA extraction and cDNA synthesis from mouse arteries

RNA was extracted from snap-frozen abdominal aorta samples from Myh11:Confetti and Myh11:Confeti:Lmna^1827T/1827T^ mice using RNeasy Plus Universal kit (QIAGEN). RNA concentration and quality were checked using Tecan NanoQuant Infinite M200 Pro. cDNA synthesis was performed using the SuperScript III (Invitrogen, Life Technologies) kit following the manufacturer’s instructions.

### Absolute quantification by ddPCR

Absolute quantification was performed using QX200 ddPCR system (Bio-Rad). 0.25–500 μg of RNA was extracted from arteries before cDNA synthesis. The primers used were Progerin Fwd: 5′-ACTGCAGCAGCTCGGGG-3′ and Rev: 5′-TCTGGGGGCTCTGGGC-3′; IL-6 Fwd: 5′-AGACAGCCACTC ACCTCTTCAG-3′ and Rev: 5′-TTCTGCCAGTGCCTCTTTG CTG-3′; TNFa Fwd: 5′-CTC TTCTGCCTGCTGCACTTTG-3′ and Rev: 5′-ATGGGCTACAGGCTTGTCAC TC-3′; Runx2 Fwd: 5′-CAGATGGGACTGTGGTTACC-3′ and Rev: 5′-TGTCTGT GCCTTCTTGGTTC-3′; Bmp2 Fwd: 5′-CACCGTGCGCAGCTTCCA-3′ and Rev: 5′-CCGGGCCGTTTTCCCACTCA-3′; Spp1 Fwd: 5′-TTGGCAGTGATTTGCTTTTG-3′ and Rev: 5′-TCTGGGTGCAGGCTGTAAA-3′; GAPDH Fwd: 5′-GAGCGA GATCCCTCCAAAAT-3′ and Rev: 5′-CATCACGCCACAGTTTCC-3′; and β-actin Fwd: 5′-CCTAGGCACCAGGGTGTGAT-3′ and Rev: 5′-CCATGTCGTCCCAG TTGGTAA-3′. The following cycling conditions were used: 5 min at 95 °C, 40 cycles of 30 s at 95 °C and 1 min at 60 °C (GAPDH, β-actin, IL-6, TNF, Runx2 and Spp1), 63 °C (progerin) or 65.6 °C (Bmp2), 5 min at 4 °C and 5 min at 90 °C. Samples were run in single wells for GAPDH, IL-6, TNF and progerin, and in duplicate for β-actin, Runx2, Spp1 and BmP2. The analysis was performed according to previously described procedures^20^. The normalized transcript levels for arterial IL-6 and TNF were transformed to arbitrary units by multiplying by 1,000.

### Elastin fiber pathology

To assess elastin fiber breaks and linearization, we took advantage of the autofluorescence of the elastin fibers. The number of elastin fiber breaks was counted in the whole medial layer. For elastin fiber linearization, we defined five random regions of interest across the artery covering 10,000 μm^2^. We counted the number of elastin coils, defined as two adjacent elastin fiber crests, present in all five regions of interest.

### Statistics and reproducibility

Human data were analyzed using nonparametric tests to assess differences between the different cohorts studied (patients with CKD, Ctrls and CVD Ctrls). The data distribution was not assumed to be normal, but this was not formally tested. These tests include Mann–Whitney *U*-test with a two-tailed 95% confidence and Kruskal–Wallis test with Dunn’s correction for multiple comparisons. Correlations were performed using Pearson correlation coefficients with a two-tailed 95% confidence interval when the sample *n* ≥ 30 or using Spearman correlation coefficients with a two-tailed 95% confidence interval when the sample *n* < 30. Mouse data were analyzed using parametric tests to assess differences between the different groups studied. The data distribution was assumed to be normal, but this was not formally tested. These tests included unpaired *t*-test with a two-tailed 95% confidence interval, multiple unpaired *t*-test with Holm–Sidak’s correction for multiple comparisons, one-way analysis of variance (ANOVA) and two-way ANOVA with Tukey’s correction for multiple comparisons. For all experiments, *P* < 0.05 was considered significant (*P < 0.05, **P < 0.005 and ***P < 0.001). The number of biological replicates and independent experimental repeats is indicated in each figure legend corresponding to each experiment. Data are shown as mean ± s.e.m. Data were collected in Microsoft Excel v.16.17 and GraphPad Prism (v.10.1.1) was used for all statistical tests for analysis of experimental results. For experiments performed on human material, samples were selected in an unbiased way, independently from covariates, but were allocated to the CKD, Ctrls or CVD Ctrls groups to establish groups with sufficient sample size for statistical analyses. Sample selection was random and based on availability. Investigators were blinded to group allocation during data collection, experimental procedures and analysis. For experiments performed on mouse tissues, all the generated mice with a genotype of interest were included in the study. Some experiments were run in a subset of samples and groups were established based on genotypes, with sufficient sample size for statistical analyses. Investigators were blinded to group allocation during data analysis. To verify the reproducibility of our findings, experiments were performed using at least three biological replicates. No data were excluded from the analyses.

### Reporting summary

Further information on research design is available in the Nature Portfolio Reporting Summary linked to this article.

## Supplementary information

Reporting Summary

Supplementary Tables 1–6 Table 1: Comparison of population frequency, phenotype and vascular pathology between CKD stage 5 (kidney failure) and HGPS. Table 2: Demographic and biochemical characteristics of patients with CKD stage 5 and controls. Table 3: Fractional abundance of the *LMNA* c.1824C>T in patients with CKD. Table 4: Fractional abundance of the *LMNA* c.1824C>T in controls. Table 5: Fractional abundance of the *LMNA* c.1824C>T in CVD controls. Table 6: Selected non-*LMNA* genetic mutations for rare event detection in PBMCs from CKD and controls.

Supplementary Video 1 Spatial reconstruction of a CKD artery.

## Source data

Source Data Fig. 1 Statistical source data for Fig. 1.

Source Data Fig. 2 Statistical source data for Fig. 2.

Source Data Fig. 3 Statistical source data for Fig. 3.

Source Data Fig. 4 Statistical source data for Fig. 4.

Source Data Fig. 5 Statistical source data for Fig. 5.

Source Data Fig. 6 Statistical source data for Fig. 6.

Source Data Fig. 7 Statistical source data for Fig. 7.

Source Data Fig. 8 Statistical source data for Fig. 8.

Source Data Extended Data Fig. 1 Statistical source data for Extended Data Fig. 1.

Source Data Extended Data Fig. 1 Unprocessed western blot.

Source Data Extended Data Fig. 2 Statistical source data for Extended Data Fig. 2.

Source Data Extended Data Fig. 3 Statistical source data for Extended Data Fig. 3.

Source Data Extended Data Fig. 4 Statistical source data for Extended Data Fig. 4.

Source Data Extended Data Fig. 5 Statistical source data for Extended Data Fig. 5.

Source Data Extended Data Fig. 6 Statistical source data for Extended Data Fig. 6.

Source Data Extended Data Fig. 7 Statistical source data for Extended Data Fig. 7.

Source Data Extended Data Fig. 8 Statistical source data for Extended Data Fig. 8.

## Data Availability

There are no restrictions on data availability in this paper. All the information is included in the paper. All main and extended data figures have associated Source Data that are provided as an Excel worksheet organized by figures, including statistics and exact *P* values. Supplementary Information is available for this paper as Supplementary Tables 1–6 and Supplementary Video 1. Source data are provided with this paper.
