# Supplementary material for: Recurrent somatic mutation and progerin expression in early vascular aging of chronic kidney disease
Source: Nat Aging. 2025 Jun 10;5(6):1046–62. doi: 10.1038/s43587-025-00882-6 (PMC12176630; doi:10.1038/s43587-025-00882-6)
Supplement: Supplementary file 1 — Reporting Summary [file 43587_2025_882_MOESM1_ESM.pdf]

Corresponding author(s): NATAGING-A07825-T
Maria Eriksson, Gwladys Revêchon

Last updated by author(s): March 7, 2025

Reporting Summary

Nature Portfolio wishes to improve the reproducibility of the work that we publish. This form provides structure for consistency and transparency in reporting. For further information on Nature Portfolio policies, see our Editorial Policies and the Editorial Policy Checklist.

Statistics

For all statistical analyses, confirm that the following items are present in the figure legend, table legend, main text, or Methods section.

n/a	Confirmed	
☐	☒	The exact sample size (n) for each experimental group/condition, given as a discrete number and unit of measurement
☐	☒	A statement on whether measurements were taken from distinct samples or whether the same sample was measured repeatedly
☐	☒	The statistical test(s) used AND whether they are one- or two-sided<br>*Only common tests should be described solely by name; describe more complex techniques in the Methods section.*
☐	☒	A description of all covariates tested
☐	☒	A description of any assumptions or corrections, such as tests of normality and adjustment for multiple comparisons
☐	☒	A full description of the statistical parameters including central tendency (e.g. means) or other basic estimates (e.g. regression coefficient) AND variation (e.g. standard deviation) or associated estimates of uncertainty (e.g. confidence intervals)
☐	☒	For null hypothesis testing, the test statistic (e.g. F, t, r) with confidence intervals, effect sizes, degrees of freedom and P value noted<br>*Give P values as exact values whenever suitable.*
☒	☐	For Bayesian analysis, information on the choice of priors and Markov chain Monte Carlo settings
☒	☐	For hierarchical and complex designs, identification of the appropriate level for tests and full reporting of outcomes
☐	☒	Estimates of effect sizes (e.g. Cohen's d, Pearson's r), indicating how they were calculated

Our web collection on statistics for biologists contains articles on many of the points above.

Software and code

Policy information about availability of computer code

Data collection	All the data was collected in Microsoft Excel version 16.17
Data analysis	DdPCR data was analyzed using QuantaSoft V.1.6; confocal images were analyzed using NIS elements Viewer and NIS Elements AR v6.02.01; statistical analysis were performed and graphs were plotted using GraphPad Prism version 6.0

For manuscripts utilizing custom algorithms or software that are central to the research but not yet described in published literature, software must be made available to editors and reviewers. We strongly encourage code deposition in a community repository (e.g. GitHub). See the Nature Portfolio guidelines for submitting code & software for further information.

Data

Policy information about availability of data

All manuscripts must include a data availability statement. This statement should provide the following information, where applicable:

- Accession codes, unique identifiers, or web links for publicly available datasets
- A description of any restrictions on data availability
- For clinical datasets or third party data, please ensure that the statement adheres to our policy

Supplementary Information is available for this paper:
Supplementary Tables 1-6
Supplementary Video 1

Research involving human participants, their data, or biological material

Policy information about studies with human participants or human data. See also policy information about sex, gender (identity/presentation), and sexual orientation and race, ethnicity and racism.

Reporting on sex and gender	Consecutive patients undergoing surgery who had given written informed consent were included in the study. Therefore gender was skewed for some sample groups (CKD: 70% males; Controls: 25% males; CVD controls: 50% males; PBMC controls: 54% males). Therefore, based on sample limitations, gender-based analyses were not performed.
Reporting on race, ethnicity, or other socially relevant groupings	Human material was obtained from live donors (CKD patients and control individuals) and analyzed independently of their race, ethnicity and other socially relevant groupings. We performed our analyses on Regional cohorts from the Stockholm area in Sweden. There are both logistic and ethical challenges in collecting large number of arterial biopsies in patients. Given the surgical protocol of kidney transplantation, we had the unique opportunity to collect arteries without risk for patients. For the purpose of our study, confounding factors were rather more related to the cause of the disease, age of the patients and patients medication, factors which have been analyzed in relation to our findings.
Population characteristics	Regional cohorts from the Stockholm area in Sweden were used. Consecutive adult CKD stage 5 (i.e. eGFR<15ml/min) patients undergoing living donor renal transplantation (LD-RTx) at the Department of Transplantation Surgery at Karolinska University Hospital were invited to participate in the study, which was approved by the regional committee of ethics in Stockholm and adhered to the statuses of the Declaration of Helsinki (Ethical Permits Dnr. 2008/1748-31/2, 2011/668-31/3, 2015/1115-31 and 244/01). All patients completed the written informed consent. CKD stage 5 patients undergoing living donor transplantation constitute a healthier selection than CKD stage 5 patients remaining on dialysis, with less CVD and less vascular calcification. Epigastric arteries were obtained from 50 CKD stage 5 patients during LD-RTx. Media calcification score of epigastric arteries from CKD patients is a good predictor of cardiovascular events and mortality, making this type of artery relevant to study CVD in the context of CKD5. Control arteries were obtained from 24 patients without history of CVD, who underwent surgery for inguinal hernia or laparoscopic cholecystectomy for gallstone disease. Removing a small piece of the artery during surgery is not an uncommon procedure, and no complications have been reported from any of the patients involved in this study. Femoral control arteries were collected from 10 deceased individuals with history of CVD (approved by the Uppsala Ethics Board Review, Dnr 2014/500/1). Blood samples were obtained from 26 of the 50 CKD stage 5 patients, and 26 controls. Basic characteristics of the CKD patients and controls are outlined in Table S1. The causes of CKD were chronic glomerulonephritis (n=14), adult polycystic kidney disease (n=8), diabetes (n=9), NUD (n=7) and other (or unknown) renal diseases (n=12). The most commonly used medications were erythropoiesis stimulating agents (n=38), active vitamin D (n=43), ACE-inhibitors and/or angiotensin receptor blockers (n=29). Prior to RTx, 13 of the patients had been diagnosed with cerebrovascular (n=2), cardiovascular (n=6), and/or peripheral vascular disease (n=5) (grouped as CVD). Out of the 50 patients, 20 patients received conservative treatment before undergoing pre-emptive LD-RTx, while 30 patients underwent dialysis treatment prior to LD-RTx for a median period of 0.5 years, by hemodialysis (HD; n=16), by peritoneal dialysis (PD; n=13) or both, as one patient who initially received HD, later switched to PD. Ages of the participants were as follow: CKD patients: 20 to 69 years (average: 46.9), Control individuals: 27 to 64 years (average: 47.8), CVD controls: 52 to 79 years (average: 63.4) and PBMC controls: 21 to 81 years (average: 48).
Recruitment	Consecutive adult CKD stage 5 (i.e. eGFR<15ml/min) patients undergoing living donor renal transplantation (LD-RTx) at the Department of Transplantation Surgery at Karolinska University Hospital were invited to participate in the study. Similarly, patients without history of CVD, who underwent surgery for inguinal hernia or laparoscopic cholecystectomy for gallstone disease were invited to participate. All patients completed the written informed consent. Every patient that agreed to participate was included in the study, no selection based on patients characteristics was made. Femoral control arteries were collected from 10 deceased individuals with history of CVD. No selection was made. Because there was no prior selection of the individuals to be included in this study, the cohorts are heterogeneous. This likely impacted the correlations between progerin expression/mutation allele frequency and age, as described in the discussion of the paper.
Ethics oversight	Ethical permits were approved by the regional committee of ethics in Stockholm and adhered to the statuses of the Declaration of Helsinki (Ethical Permits Dnr. 2008/1748-31/2, 2011/668-31/3, 2015/1115-31 and 244/01), and the Uppsala Ethics Board Review (Dnr 2014/500/1).

Note that full information on the approval of the study protocol must also be provided in the manuscript.

Field-specific reporting

Please select the one below that is the best fit for your research. If you are not sure, read the appropriate sections before making your selection.

☒ Life sciences ☐ Behavioural & social sciences ☐ Ecological, evolutionary & environmental sciences

For a reference copy of the document with all sections, see nature.com/documents/nr-reporting-summary-flat.pdf

Life sciences study design

All studies must disclose on these points even when the disclosure is negative.

Sample size	Sample size was determined based on sample availability, and so not determined by a statistical method. There are both logistic and ethical challenges in collecting large number of arterial biopsies in CKD patients. Given the surgical protocol of kidney transplantation, we had the unique opportunity to collect arteries without risk for patients. The sample size of the control cohort is rather small because we use regional cohorts from the Stockholm area in Sweden, matching the regional cohort of CKD samples, and it is difficult to obtain arterial samples from healthy individuals to match our CKD population.

	The sample size of mice generated for this study is sufficient to answer our questions and perform statistical analyses, taking into account the challenges of generating those cohorts and ethical recommendations.
Data exclusions	No data or samples were excluded from the experimental analysis.
Replication	With measurements from individual biological replicates (minimum n=3) . All attempts of replication were successful with the indicated biological replicates in the manuscript.
Randomization	For experiments that do not include the full set of CKD, control and CVD control samples, sample selection was random and based on material availability.<br>All mice generated with a genotype of interest were included in the study. Some experiments were run in a subset of samples and groups were established based on genotypes, with sufficient sample size for statistical analyses.
Blinding	For each experiment performed on human material, samples were selected in an unbiased way, independently from covariates, but were allocated to the CKD, control or CVD control groups, in order to establish groups that contain sufficient sample size for statistical analysis. However, the investigators were blinded to group allocation during data collection, experimental procedures and analysis.<br>For experiments performed on mouse tissues, all the mice generated with a genotype of interest were included in the study, and the investigators were blinded to group allocation during data analysis.

Reporting for specific materials, systems and methods

We require information from authors about some types of materials, experimental systems and methods used in many studies. Here, indicate whether each material, system or method listed is relevant to your study. If you are not sure if a list item applies to your research, read the appropriate section before selecting a response.

Materials & experimental systems

n/a	Involved in the study	
☐	☒	Antibodies
☐	☒	Eukaryotic cell lines
☒	☐	Palaeontology and archaeology
☐	☒	Animals and other organisms
☒	☐	Clinical data
☒	☐	Dual use research of concern
☒	☐	Plants

Methods

n/a	Involved in the study	
☒	☐	ChIP-seq
☒	☐	Flow cytometry
☒	☐	MRI-based neuroimaging

Antibodies

Antibodies used	anti-human progerin (mAb, 1:150, clone 13A4, #ALX-804-662-R200, Enzo Life Science)<br>anti-CD31 (pAb, ab28364, Abcam)<br>anti-human prelamin A (pAb, C-20, sc-6214, Santa Cruz Biotechnology)<br>anti-Ki67 (mAb, 1:150, clone MM1, VP-452, Vector Laboratories)<br>anti-Ki67 (pAb, ab15580, Abcam)<br>anti-PCNA (pAb, ab18197, Abcam)<br>anti-53BP1 (pAb, ab36823, Abcam)<br>anti-P21 Waf1/Cip1 (mAb, 1:200, F-5, sc-6246, Santa Cruz Biotechnology)<br>anti-P16 (mAb, 1:1000, ab54210, Abcam)<br>anti-phospho-p53 (pAb, Ser20, 9287, Cell Signaling)<br>anti-actin alpha-smooth muscle-Cy3 (mAb, 1:3000, clone 1A4, C6198, Sigma-Aldrich)<br>anti-lamin A/C (pAb, N-18, sc-6215, Santa Cruz Biotechnologies)<br>and beta-actin (mAb, 1:5000, AC-15, A5441, Sigma-Aldrich)<br>anti-ATR (pAb, phospho Thr1989, GTX128145, GeneTex)<br>anti-phospho-histone H2AX (mAb, 1:150, Ser139, clone JBW301, 05-636, Merck Millipore)<br>anti-GRP78 BiP (pAb, ab21685, Abcam)<br>anti-BiP (mAb, 1:200, C50B12, 3177, Cell Signaling)<br>goat anti-rabbit IgG (H+L) Alexa Fluor 488 (A11034, Invitrogen)<br>goat anti-mouse IgG (H+L) Alexa Fluor 555 (A21422, Invitrogen)<br>rabbit anti-goat IgG (H+L) Alexa Fluor 633 (A21086, Invitrogen)
Validation	anti-human progerin (mAb, clone 13A4, #ALX-804-662-R200, Enzo Life Science)<br>Cited by: Viceconte, McKenna, Eriksson. Low levels of the reverse transactivator fail to induce target transgene expression in vascular smooth muscle cells. PLoS ONE. 9:e104098 (2014).<br><br>anti-CD31 (pAb, ab28364, Abcam)<br>Cited by: Gong et al. The pentose phosphate pathway mediates hyperoxia-induced lung vascular dysgenesis and alveolar simplification in neonates. JCI Insight 6:e137594 (2021).<br><br>anti-human prelamin A (pAb, C-20, sc-6214, Santa Cruz Biotechnology)<br>Cited by: Ragnauth et al. Prelamin A acts to accelerate smooth muscle cell senescence and is a novel biomarker of human vascular aging. Circulation 121(20):2200-10 (2010).

anti-ki67 (mAb, clone MM1, VP-452, Vector Laboratories)
Cited by: Revêchon et al. Rare progerin-expressing preadipocytes and adipocytes contribute to tissue depletion over time. Sci Rep 7(1):4405 (2017).

anti-Ki67 (pAb, ab15580, Abcam)
Cited by: Labbé et al. Angiopoietin-like 2 is essential to aortic valve development in mice. Commun Biol 5(1):1277 (2022).

anti-PCNA (pAb, ab18197, Abcam)
Cited by: Hengel et al. Loss-of-function mutations in UDP-Glucose 6-Dehydrogenase cause recessive developmental epileptic encephalopathy. Nat Commun 11:595 (2020).

anti-53BP1 (pAb, ab36823, Abcam)
Cited by: Watanabe et al. Age-related dysfunction of the DNA damage response in intestinal stem cells. Inflamm Regen 39:8 (2019).

anti-P21 Waf1/Cip1 (mAb, F-5, sc-6246, Santa Cruz Biotechnology)
Cited by: Ryu et al. The Bcl-2/Bcl-xL inhibitor ABT-263 attenuates retinal degeneration by selectively inducing apoptosis in senescent retinal pigment epithelial cells. Mol Cell 46(7):420-429 (2023).

anti-P16 (mAb, ab54210, Abcam)
Cited by: Safwan-Zaiter et al. Dynamic spatiotemporal expression pattern of the senescence-associated factor p16Ink4a in development and aging. Cells 11(3):541 (2022).

anti-phospho-p53 (pAb, Ser20, 9287, Cell Signaling)
Cited by: Bar et al. Association between p53 protein phosphorylated at Serine 20 expression and ovarian carcinoma stem cells phenotype: correlation with clinicopathological parameters of ovarian cancer. Neoplasma 66(5):801-809 (2019).

anti-actin alpha-smooth muscle-Cy3 (mAb, clone 1A4, C6198, Sigma-Aldrich)
Cited by: Cosgrove et al. Lysyl oxidase like-2 contributes to renal fibrosis in Col4α3/Alport mice. Kidney Int. 94(2):303-14 (2018).

lamin A/C (pAb, N-18, sc-6215, Santa Cruz Biotechnologies)
Cited by: Sagelius et al. Targeted transgenic expression of the mutation causing Hutchinson-Gilford progeria syndrome leads to proliferative and degenerative epidermal disease. J Cell Sci 121:969:78 (2008).

and beta-actin (mAb, AC-15, A5441, Sigma-Aldrich)
Cited by: Morita et al. Evaluation of zinc (II) chelators for inhibiting p53-mediated apoptosis. Oncotarget 4:2439:50 (2013).

anti-ATR (pAb, phospho Thr1989, GTX128145, GeneTex)
Cited by: Watanabe et al. Age-related dysfunction of the DNA damage response in intestinal stem cells. Inflamm Regen 39:8 (2019).

anti-phospho-histone H2AX (mAb, Ser139, clone JBW301, 05-636, Merck Millipore)
Cited by: Rosengardten et al. Stem cell depletion in Hutchinson-Gilford progeria syndrome. Aging Cell 10(6):1011-20 (2011).

anti-GRP78 BiP (pAb, ab21685, Abcam)
Cited by: Suh et al. Restoration of visual function in adult mice with an inherited retinal disease via adenine base editing. Nat Biomed Eng 5(2):169-78 (2021).

anti-BiP (mAb, 3177, Cell Signaling)
Cited by: Hamczyk et al. Progerin accelerates atherosclerosis by inducing endoplasmic reticulum stress in vascular smooth muscle cells. EMBO Mol Med 11(4):e9736 (2019).

Eukaryotic cell lines

Policy information about cell lines and Sex and Gender in Research

Cell line source(s)	Control and HGPS Human B-lymphoblasts were obtained from the Coriell BioBank (Ctrl: AG03504, HGPS: AG03506, AG10587)<br>Control and HGPS Human dermal fibroblasts were obtained from the Progeria Research Foundation (Ctrl: HGADFN168, HGPS: HGADFN003)<br>HGPS Human induced pluripotent stem cells were obtained from the Progeria Research Foundation (HGADFN003 iPS1B)<br>Human primary aortic smooth muscle cells were obtained from ATCC (PCS-100-012).
Authentication	Control: AG03504 and HGADFN168<br>Authentication: By STR profiling, sequencing and mutation specific PCR primer amplification for the LMNA c.1824C>T transition Eriksson et al. Nature 423: 293 (2003)<br>HGPS: AG03506, AG10587 and HGADFN003<br>Authentication: By STR profiling, sequencing and mutation specific PCR primer amplification for the LMNA c.1824C>T transition Eriksson et al. Nature 423: 293 (2003)
Mycoplasma contamination	All cells are negative for mycoplasma.
Commonly misidentified lines (See ICLAC register)	No commonly misidentified cell lines were used in the study.

Animals and other research organisms

Policy information about studies involving animals; ARRIVE guidelines recommended for reporting animal research, and Sex and Gender in Research

Laboratory animals	Myh11-CreERT2 mice were ordered from The Jackson Laboratory38. LmnaLCS (carrying the murine HGPS mutation Lmna c.1827C>T) and R26R-Confetti mice were a kind gift from Dr. Vicente Andrés and Dr. Pekka Katajisto, respectively. All mice were maintained on a C57Bl6 background. Whole litters were injected intraperitoneally with tamoxifen on three consecutive days starting at P3 or, adult mice of genotype of interest were injected intraperitoneally with tamoxifen on five consecutive days, starting betwwen week 5 and week 13. Mice were sacrificed, collected and aortas were analyzed either at P8 or at P21 (for pups), or 2- to 10 weeks post-tamoxifen injections (for adults).<br>Wild-type C57Bl6 mice were used at P3, P5, P8, P14 and P21 to assess the proliferation capacity of VSMCs in the aorta.
Wild animals	The study did not involve wild animals.
Reporting on sex	Mice were crossed to generate Myh11:Confetti, Myh11:Confetti:Lmna1827T/+ and Myh11:Confeti:Lmna1827T/1827T. Given that the Myh11-CreERT2 BAC transgene is inserted in the Y chromosome, only males were used in the study, and so, no sex-based analysis was performed.<br>To assess proliferation of VSMCs in the aorta of wild-type mice, both males and females were randomly included in the analysis, which did not interfere with the results obtained. Proliferation data obtained from wild-type, Myh11:Confetti, Myh11:Confetti:Lmna1827T/+ and Myh11:Confeti:Lmna1827T/1827T was not different, suggesting that the sex of the animals included in the study should not impact the results obtained.
Field-collected samples	The study did not involve field-collected samples
Ethics oversight	Animal studies were approved by Linköping's regional animal research ethical review board (Dnr. 6088-2020). All procedures were performed in accordance with the institutional guidelines and regulations.

Note that full information on the approval of the study protocol must also be provided in the manuscript.

Plants

Seed stocks	*Report on the source of all seed stocks or other plant material used. If applicable, state the seed stock centre and catalogue number. If plant specimens were collected from the field, describe the collection location, date and sampling procedures.*
Novel plant genotypes	*Describe the methods by which all novel plant genotypes were produced. This includes those generated by transgenic approaches, gene editing, chemical/radiation-based mutagenesis and hybridization. For transgenic lines, describe the transformation method, the number of independent lines analyzed and the generation upon which experiments were performed. For gene-edited lines, describe the editor used, the endogenous sequence targeted for editing, the targeting guide RNA sequence (if applicable) and how the editor was applied.*
Authentication	*Describe any authentication procedures for each seed stock used or novel genotype generated. Describe any experiments used to assess the effect of a mutation and, where applicable, how potential secondary effects (e.g. second site T-DNA insertions, mosiacism, off-target gene editing) were examined.*
