# Supplementary figures and images for: Recurrent somatic mutation and progerin expression in early vascular aging of chronic kidney disease
Source: Nat Aging. 2025 Jun 10;5(6):1046–62. doi: 10.1038/s43587-025-00882-6 (PMC12176630; doi:10.1038/s43587-025-00882-6)

Source data: Western blots of B-actin, N18 and Progerin

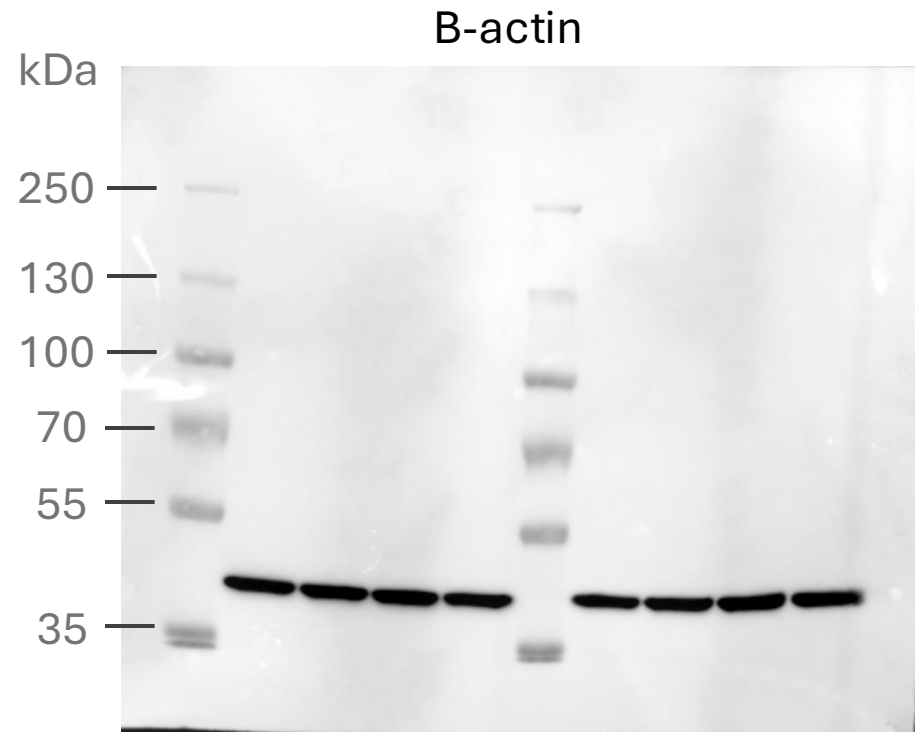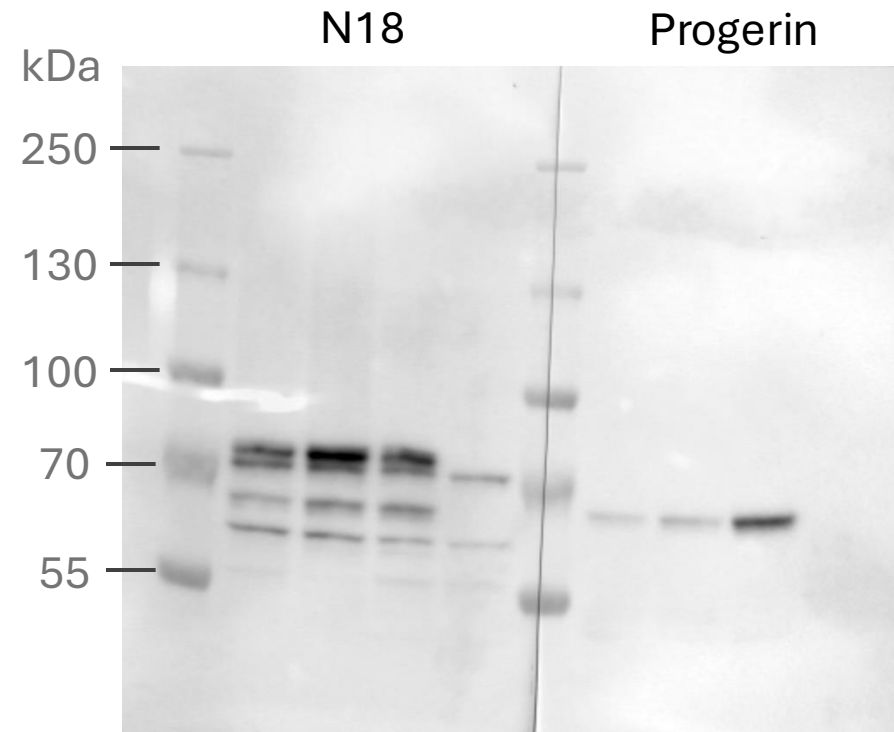

Supplement: Supplementary file 13 — Unprocessed western blot. [file 43587_2025_882_MOESM13_ESM.pdf]
